# Methodological Frameworks and Dimensions to Be Considered in Digital Health Technology Assessment: Scoping Review and Thematic Analysis

**DOI:** 10.2196/48694

**Published:** 2024-04-10

**Authors:** Joan Segur-Ferrer, Carolina Moltó-Puigmartí, Roland Pastells-Peiró, Rosa Maria Vivanco-Hidalgo

**Affiliations:** 1 Agency for Health Quality and Assessment of Catalonia Barcelona Spain

**Keywords:** digital health, eHealth, mHealth, mobile health, AI, artificial intelligence, framework, health technology assessment, scoping review, technology, health care system, methodological framework, thematic analysis

## Abstract

**Background:**

Digital health technologies (dHTs) offer a unique opportunity to address some of the major challenges facing health care systems worldwide. However, the implementation of dHTs raises some concerns, such as the limited understanding of their real impact on health systems and people’s well-being or the potential risks derived from their use. In this context, health technology assessment (HTA) is 1 of the main tools that health systems can use to appraise evidence and determine the value of a given dHT. Nevertheless, due to the nature of dHTs, experts highlight the need to reconsider the frameworks used in traditional HTA.

**Objective:**

This scoping review (ScR) aimed to identify the methodological frameworks used worldwide for digital health technology assessment (dHTA); determine what domains are being considered; and generate, through a thematic analysis, a proposal for a methodological framework based on the most frequently described domains in the literature.

**Methods:**

The ScR was performed in accordance with the guidelines established in the PRISMA-ScR guidelines. We searched 7 databases for peer reviews and gray literature published between January 2011 and December 2021. The retrieved studies were screened using Rayyan in a single-blind manner by 2 independent authors, and data were extracted using ATLAS.ti software. The same software was used for thematic analysis.

**Results:**

The systematic search retrieved 3061 studies (n=2238, 73.1%, unique), of which 26 (0.8%) studies were included. From these, we identified 102 methodological frameworks designed for dHTA. These frameworks revealed great heterogeneity between them due to their different structures, approaches, and items to be considered in dHTA. In addition, we identified different wording used to refer to similar concepts. Through thematic analysis, we reduced this heterogeneity. In the first phase of the analysis, 176 provisional codes related to different assessment items emerged. In the second phase, these codes were clustered into 86 descriptive themes, which, in turn, were grouped in the third phase into 61 analytical themes and organized through a vertical hierarchy of 3 levels: level 1 formed by 13 domains, level 2 formed by 38 dimensions, and level 3 formed by 11 subdimensions. From these 61 analytical themes, we developed a proposal for a methodological framework for dHTA.

**Conclusions:**

There is a need to adapt the existing frameworks used for dHTA or create new ones to more comprehensively assess different kinds of dHTs. Through this ScR, we identified 26 studies including 102 methodological frameworks and tools for dHTA. The thematic analysis of those 26 studies led to the definition of 12 domains, 38 dimensions, and 11 subdimensions that should be considered in dHTA.

## Introduction

### Background

Digital health technologies (dHTs) are driving the transformation of health care systems. They are changing the way in which health services are delivered, and showing great potential to address some of the major challenges that European health systems, including the Spanish National Health System (SNS), are facing, such as the progressive aging of the population [1,2]; the growing demand for health and long-term care services [2]; the rise in health care costs, increasing financial pressures on health and welfare systems [1,3]; and the unequal distribution of health services across different geographical regions [4,5]. In addition, dHT can improve the accessibility, sustainability, efficiency, and quality of health care systems [6,7], leading to their becoming a determinant of health on their own [6,8].

However, the digital transformation of health care systems and the implementation of dHT (eg, artificial intelligence [AI]–based solutions, data-driven health care services, or the internet of things) are slow and unequal across different European regions [9,10]. Some of the reasons for this are (1) the immaturity of regulatory frameworks for the use of dHTs [9], (2) the lack of funding and investment for the implementation of dHTs [9], (3) the lack of sufficient and appropriate infrastructures and common standards for data management [6,9], (4) the absence of skills and expertise of professionals and users [10], and (5) the scarcity of strong evidence regarding the real benefits and effects of dHTs on health systems and people’s well-being, as well as the cost-effectiveness of these technologies. This makes decision-making difficult, potentially leading to the development and reproduction of low-value and short-lived dHTs [6,11].

To overcome these challenges, harness the potential of dHTs, and avoid nonintended consequences, the World Health Organization (WHO) [4,11] states that dHTs should be developed under the principles of transparency, accessibility, scalability, privacy, security, and confidentiality. Their implementation should be led by robust strategies that bring together leadership, financial, organizational, human, and technological resources, and decisions should be guided by the best-available evidence [4,11].

Regarding this last aspect, health technology assessment (HTA), defined as a “multidisciplinary process that uses explicit methods to determine the value of a health technology at different points in its life cycle,” is a widely accepted tool to inform decision-making and promote equitable, efficient, and high-quality health systems [12,13].

Generally, HTA is conducted according to specific methodological frameworks, such as the HTA Core Model of the European Network for Health Technology Assessment (EUnetHTA) [14] and the guidelines for the development and adaptation of rapid HTA reports of the Spanish Network of Agencies for Assessing National Health System Technologies and Performance (RedETS) [15]. These frameworks establish the methodologies to follow and the elements to evaluate. Although these frameworks are helpful instruments for evaluating various health technologies, they have certain limitations in comprehensively assessing dHTs. For this reason, in the past few years, different initiatives have emerged to adapt existing methodological frameworks or develop new ones. The objective is to consider additional domains (eg, interoperability, scalability) to cover the intrinsic characteristics of dHTs [16-18]. Examples of these initiatives are the Evidence Standard Framework (ESF) of National Institute for Health and Care Excellence (NICE) [19] or the Digi-HTA Framework of the Finnish Coordinating Center for Health Technology Assessment (FinCCHTA) [16]. Nonetheless, the majority of these frameworks have certain constraints, such as being designed for a particular socioeconomic or national setting, which restricts their transferability or suitability for use in other countries; the specificity or exclusion of certain dHTs, resulting in limitations in their application; or the limited evidence regarding their actual usefulness.

In this context, we performed a scoping review (ScR) with the aim of identifying the methodological frameworks that are used worldwide for the evaluation of dHTs; determining what dimensions and aspects are considered for each type of dHT; and generating, through a thematic analysis, a proposal for a methodological framework that is based on the most frequently described dimensions in the literature. This research focused mainly on mobile health (mHealth), non–face-to-face care models and medical devices that integrate AI, as these particular dHTs are the ones most frequently assessed by HTA agencies and units of RedETS.

### Identifying Research Questions

This ScR followed by a thematic analysis answered the following research questions:

- What methodological frameworks currently exist for digital health technology assessment (dHTA)?
- What domains and dimensions are considered in dHTA?
- Do the different domains and dimensions considered depend on whether the dHT addressed is a non–face-to-face care model of health care provision, a mobile device (mHealth), or a device that incorporates AI?

## Methods

### Overview of Methods for Conducting the Scoping Review

We conducted an ScR of the literature and a thematic analysis of the studies included according to the published protocol [20]. The ScR aimed to answer the first research question, while the thematic analysis aimed to answer the second and third research questions. Spanish experts from various domains of HTA and dHT collaborated throughout the study design and development.

The ScR of the available scientific literature was carried out in accordance with the PRISMA-ScR (Preferred Reporting Items for Systematic Reviews and Meta-Analysis extension for Scoping Reviews) guidelines (Multimedia Appendix 1) [21] and following the recommendations of Peters et al [22] and Pollock et al [23].

### Ethical Considerations

As this work was an ScR, no ethical board approval was required.

### Search Strategy

The search strategy (Multimedia Appendix 2) was designed by an experienced information specialist (author RP-P) in accordance with the research questions and using the validated filter of Ayiku et al [24] for health apps, adding the terms for concepts related to mHealth, remote care models, AI, digital health, methodological frameworks, and HTA. The strategy was peer-reviewed according to the “Peer Review of Electronic Search Strategies Statement” [25] by authors JS-F and CM-P and was executed in the following 7 databases, considering the characteristics of each in terms of syntax, controlled vocabulary, and proximity operators: Medline (OVID), CINAHL Plus, Embase, Cochrane Library, Scopus, Web of Science, and TripDatabase. Note that no time, language, or other filters were used.

The identification of relevant studies was complemented with a manual search based on the references in the included studies, as well as the websites of the HTA agencies identified through the web pages of EUnetHTA, the International Network for Agencies for Health Technology Assessment (INAHTA), and Health Technology Assessment International (HTAi). Additionally, a search was conducted in Google Scholar, limiting the results to the first 250 items in order to guarantee the inclusion of all pertinent studies [26].

### Inclusion and Exclusion Criteria

The inclusion criteria used in the reference-screening process were based on the previously detailed research questions and are outlined in Textbox 1 using the Population/Problem, Phenomenon of Interest, Context and Design (PICo-D) format [27,28]. The PICo-D format was used instead of the traditional Population/Problem, Intervention, Comparator, Outcomes, Design (PICO-D) format due to the qualitative nature of the research questions and the characteristics of the phenomenon of interest.

Studies were excluded if they were published before 2011, due to the rapid evolution of dHTs in the past few years, did not describe dimensions or evaluation criteria, or were based on methodological frameworks not intended for the assessment of dHTs (eg, EUnetHTA Core Model 3.0). Likewise, we excluded comments, editorials, letters, conference abstracts, frameworks, or tools focusing on the evaluation of dHTs by users (eg, User version of Mobile App Rating Scale [uMARS]) or documents in languages other than English, Spanish. or Catalan.

**Research questions in Population/Problem, Phenomenon of Interest, Context and Design (PICo-D) format.**

**Population/problem**

Digital health technology assessment (dHTA)

**Phenomenon of interest**

Specific methodological frameworks for the evaluation of digital health (with special focus on mobile health [mHealth]: non–face-to-face care models and medical devices that integrate artificial intelligence [AI] due the type of technologies mostly assessed in the Spanish National Health System [SNS]) that describe the domains to be evaluated in dHTA

**Context**

Health technology assessment (HTA)

**Design**

Methodological guidelines and frameworks, scoping reviews (ScRs), systematic reviews (SRs), consensus documents, and qualitative studies

### Reference Screening and Data Extraction

The screening of studies was carried out by authors CM-P and JS-F in 2 phases in accordance with the selection criteria detailed earlier (Textbox 1) and in a single-blind peer review manner. The first phase consisted of screening of the titles and abstracts of the studies identified in the bibliographic search. The second phase consisted of full-text screening of the studies included in the previous phase.

Data extraction was performed by 3 authors (CM-P, RP-P, and JS-F) using the web and desktop versions of ATLAS.ti version 22.0 (Scientific Software Development GmbH) [29] and the data extraction sheets designed ad hoc for this purpose following the recommendations of the *Cochrane Handbook for Systematic Reviews of Interventions* [30].

When disagreements emerged in either of the 2 processes, a consensus was reached between the 3 reviewers (CM-P, RP-P, and JS-F). When a consensus was not possible, a fourth reviewer (author RMV-H) was consulted.

### Collecting, Summarizing, and Reporting the Results

A descriptive analysis was carried out to evaluate and report the existing methodological frameworks and their characteristics.

#### Overview of Methods for Thematic Analysis

The thematic analysis was performed following the recommendations and phases described by Thomas and Harden [31] to determine HTA dimensions for dHTs: (1) line-by-line text coding, (2) development of descriptive topics, and (3) generation of analytical themes. Both analyses were carried out by 3 authors (CM-P, RP-P, and JS-F) using the web and desktop versions of ATLAS.ti version 22.0 [29].

Dimensions identified from systematic reviews (SRs) that were derived from primary studies also identified in our systematic search were only counted once in order to avoid duplication of data and risk of bias. It is worth mentioning that the primary studies included in the SRs were not directly analyzed but were analyzed through the findings reported in the SRs.

## Results

### Study Selection and Characteristics

A total of 3042 studies were retrieved throughout the systematic (n=3023, 99.4%) and the manual (n=19, 0.6%) search. Of these, 2238 (73.6%) studies were identified as unique after removing duplicates.

**Figure 1:**
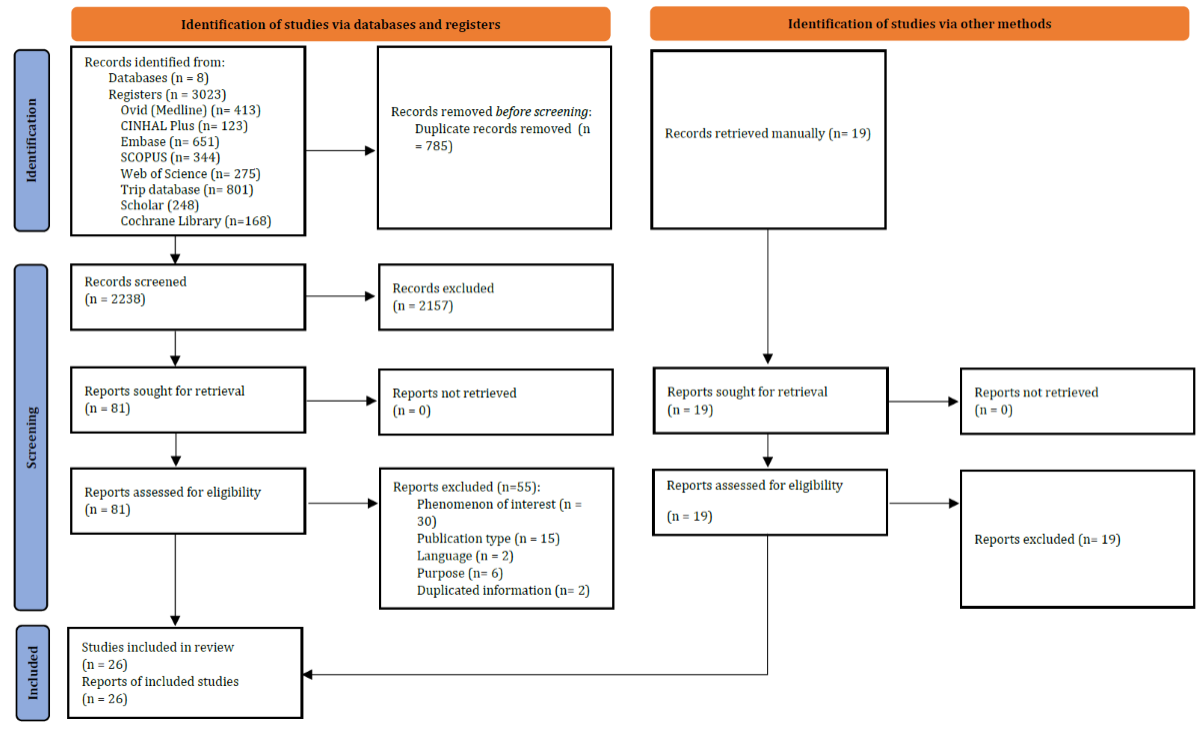
PRISMA 2020 flow diagram of the search and study selection process for new SRs, meta-analyses, and ScRs. PRISMA: Preferred Reporting Items for Systematic Reviews and Meta-Analysis; ScR: scoping review; SR: systematic review.

After title and abstract review, 81 (3.6%) studies were selected for full-text review, of which 26 (32.1%) were finally included in the analysis. The excluded studies and reasons for exclusion are detailed in Multimedia Appendix 3; in brief, the reasons for exclusion were phenomenon of interest (n=30, 37%), type of publication (n=15, 18.5%), purpose (n=6, 7.4%), language (n=2, 2.5%), and duplicated information (n=2, 2.5%). The study selection process is outlined in Figure 1 [32].

Of the 26 (32.1%) studies included in this ScR, 19 (73.1%) were designed as specific methodological frameworks for dHTA [16,17,33-47], 4 (15.4%) were SRs [48-51], 1 (3.9%) was a report from the European mHealth Hub’s working group on mHealth assessment guidelines [52], 1 (3.9%) was a qualitative study [53], and 1 (3.9%) was a viewpoint [54]. In addition, 3 (11.5%) focused on the assessment of non–face-to-face care models [33-35], 8 (30.8%) on mHealth assessment [36-40,52,53,55], 2 (7.7%) on the assessment of AI technology [41,54], 4 (15.4%) on eHealth [42,43,48,50], and 9 (34.6%) on the overall assessment of digital health [16,17,44-47,49,51,56].

### Research Question 1: Description of Identified Frameworks for dHTA

The 19 methodological frameworks for dHTA [16,17,33-47] were from various countries: The majority (n=5, 26.3%) originated in Australia [17,34,38,41,46], followed by 3 (15.8%) from the United States [43,45,56] and 2 (10.5%) from Switzerland [47,55]; the remaining 9 (47.4%) frameworks were developed in Afghanistan [42], Denmark [33], Scotland [35], Finland [16], Ireland [36], Israel [40], the United Kingdom [37], Spain [39], and Sweden [44].

The 19 methodological frameworks focused on evaluating various types of technologies. Specifically, 3 (15.8%) of them were designed for assessing non–face-to-face care models [33-35], 6 (31.6%) for mHealth [36-40], and 1 (5.3%) for AI solutions [41]. The other 9 (47.4%) frameworks addressed eHealth [42,43,56] or digital health in general [16,17,44-47], which encompasses non–face-to-face care models, mHealth, and occasionally AI-based solutions [18] within its scope. It is pertinent to mention that the differentiation between the methodological frameworks designed for the evaluation of eHealth and those designed for dHTA was based on the specific terminology and descriptions used by the authors of those frameworks.

The structures and characteristics of the analyzed methodological frameworks were considered heterogeneous in terms of evaluation specificity (whether they focused on a global evaluation that encompassed more than 1 domain or dimension or on a specific assessment that addressed only 1 domain or dimension), assessment approach (whether they adopted a phased evaluation, a domain evaluation, or a hybrid of both), and number of domains included. Regarding evaluation specificity, 17 (89.5%) methodological frameworks were classified as global as they covered various aspects or domains within their scope [16,17,33-36,38-47,55,56], while 2 (10.5%) were classified as specific as they concentrated exclusively on 1 element or domain of assessment [37,46]. Regarding the assessment approach, 14 (73.7%) methodological frameworks proposed a domain-based evaluation [16, 17, 33, 35, 36, 38-40, 43, 44, 46, 55, 56], while 4 (21.1%) proposed a hybrid one (phased and domain based) [41,42,45,47]; the remaining methodological framework did not fit into any of the previous categories, as it was not structured by domains or phases but by types of risk [37]. Finally, the number of evaluation domains considered ranged from 1 to 14, with an average of 7. Table 1 outlines the primary features of the included methodological frameworks and provides a thorough breakdown of the domains and dimensions they address.

In contrast, from 3 (75%) [49-51] of the 4 SRs [48-51] and the report from the working group on guidelines for the evaluation of mHealth solutions from the European mHealth Hub [52], we identified other methodological frameworks and tools focusing on the assessment of dHTs. Specifically, we identified 16 methodological frameworks or tools focusing on the evaluation of non–face-to-face care models [57-72], along with 37 for the evaluation of mHealth [10,52,73-95], 11 for the evaluation of eHealth [96-107], and 17 for the evaluation of dHTs in general [108-124]. Additionally, 5 (26.3%) [33,34,36,37,42] of the 19 methodological frameworks included in this ScR were also identified and analyzed in 1 or more of the 4 literature synthesis documents [49-52]. It is important to note that the difference between the frameworks we retrieved through our systematic search and those identified in the 4 SRs is the result of the narrower perspective we adopted, focusing exclusively on frameworks directly relevant to the HTA field, in line with the aims of our study. In Multimedia Appendix 4, we provide a more detailed explanation of the methodological frameworks included in the studies mentioned earlier [19,49-52,57-73,75-135].

**Table 1:** Methodological frameworks (N=19) included in this ScR^a^.

Methodological framework, year			Country		Assessment specificity		Assessment approach		Assessment domains, n
**Methodological frameworks focusing on the assessment of non–face-to-face care models (n=3, 15.8%)**									
	Model for Assessment of Telemedicine Applications (MAST), 2012 [33]	Denmark		Overall evaluation		By domain		Health problem and application description; security; clinical effectiveness; patient perspective; economic aspects; organizational aspects; sociocultural, ethical, and legal aspects (n=7)	
	Scottish Centre for Telehealth & Telecare (SCTT) Toolkit [35]	Scotland		Overall evaluation		By domain		User benefits/costs; benefits/service costs; user experience; increased use of the technology platform available to support routine local services; confidence in the use and awareness of staff; awareness of telehealth and telecare as tools (n=6)	
	Telehealth framework, 2014 [34]	Australia		Overall evaluation		By domain		Health domain; health services; communication technologies; environment configuration; socioeconomic evaluation (n=5)	
**Methodological frameworks focusing on the evaluation of mHealth^b^ technologies (n=6, 31.9%)**									
	Caulfield’s evaluation framework, 2019 [36]	Ireland		Overall evaluation		By domain		Context information; cost information; normative compliance; scientific evidence; human factors; data collection and interpretation (n=6)	
	mHealth-based technology assessment for mobile apps, 2020 [39]	Spain		Overall evaluation		By domain		General information about the clinical condition and about the mHealth solution; privacy and security; technological aspects and interoperability; evidence and clinical effectiveness; user experience, usability, acceptability, ease of use, and aesthetics; costs and economic evaluation; impact on the organization (n=7)	
	Henson’s app evaluation framework, 2019 [40]	Israel		Overall evaluation		By domain		Context information; privacy/security; scientific evidence; usability; data integration (n=5)	
	Lewis’s assessment risk framework, 2014 [37]	United Kingdom		Risk/safety assessment		N/A^c^		Risk (n=1)	
	Mobile medical app evaluation module, 2020 [38]	Australia		Overall evaluation		By domain		Description and technical characteristics; current use of technology; effectiveness; security; effectivity cost; organizational aspects; ethical aspects; legal aspects; postmarket monitoring; social aspects (n=10)	
	Vokinger, 2020 [55]	Swiss		Overall evaluation		By domain		Purpose; usability; information accuracy; organizational reputation; transparency; privacy; self-determination or user control (n=7)	
**Methodological frameworks focusing on the assessment of solutions based on AI^d^ (n=1, 5.3%)**									
	Translational Evaluation of Healthcare AI (TEHAI), 2021 [41]	Australia		Overall evaluation		Hybrid		Ability; utility; adoption (n=3)	
**Methodological frameworks focusing on the evaluation of eHealth technologies (n=3, 15.8%)**									
	Health Information Technology Evaluation Framework (HITREF), 2015 [43]	United States		Overall evaluation		By domain		Structural quality; quality of information logistics; unintended consequences/benefits; effects on quality-of-care outcomes; effects on process quality (n=5)	
	Heuristic evaluation of eHealth interventions, 2016 [56]	United States		Overall evaluation		By domain		Usability/ease of use/functionality; aesthetics; security; content; adherence; persuasive design; research evidence; owner credibility (n=8)	
	Khoja-Durrani-Sajwani (KDS) Framework, 2013 [42]	Afghanistan, Canada, Kenya, Pakistan		Overall evaluation		Hybrid		Health service results; technology results; economic results; sociotechnical and behavioral results; ethical results; preparation and change results; results of the regulation (n=7)	
**Methodological frameworks focusing on the evaluation of dHTs^e^ (n=6, 31.6%)**									
	Deontic accountability framework, 2019 [46]	Australia		Ethical evaluation		By domain		Ethical principles (n=1)	
	Digi HTA, 2019 [16]	Finland		Overall evaluation		By domain		Company information; product information; technical stability; costs; effectiveness; clinical safety; data protection and security; usability and accessibility; interoperability; AI; robots (n=11)	
	Digital Health Scorecard, 2019 [45]	United States		Overall evaluation		Hybrid		Technical validation; clinical validation; usability; costs (n=4)	
	Framework for the design and evaluation of digital health interventions (DEDHI), 2019 [44]	Sweden		Overall evaluation		By domain		Easy to use; content quality; privacy and security; responsibility; adherence; aesthetics; perceived benefits; effectiveness; quality of service; personalization; perceived enjoyment; ethics; security (n=13)	
	Monitoring and Evaluating Digital Health Interventions Guide, 2016 [47]	Swiss		Overall evaluation		Hybrid		Costs; feasibility; usability; effectiveness; implementation science; efficiency; quality; use (n=8)	
	Precision Health Applications Evaluation Framework, 2021 [17]	Australia		Overall evaluation		By domain		Novelty; adaptability; information management; performance; clinical effectiveness; quality assurance (n=6)	

^a^ScR: scoping review.

^b^mHealth: mobile health.

^c^N/A: not applicable.

^d^AI: artificial intelligence.

^e^dHT: digital health technology.

### Research Question 2: Domains and Dimensions Being Considered in dHTA

The 26 (32.1%) studies included encompassed a broad range of items to consider in dHTA and often used diverse expressions for analogous concepts. We reduced this heterogeneity through our thematic analysis according to the recommendations and phases described by Thomas and Harden [31].

In this sense, in the first phase of thematic analysis, we identified and coded 176 units of meaning (coded as provisional codes) that represented different items (domains or dimensions) of the assessment. These units were then grouped into 86 descriptive themes (second phase), which were further refined into 61 analytical themes that captured the key concepts and relationships between them (third phase). Lastly, the 61 analytical themes were arranged in a 3-level vertical hierarchy based on the evidence: level 1 (12 domains), level 2 (38 dimensions), and level 3 (11 subdimensions). We used the term “domain” to refer to a distinct area or topic of evaluation that is integral to the assessment of the technology in question. A domain may encompass multiple related concepts or dimensions that are relevant to the evaluation. Each dimension, in turn, represents a specific aspect of evaluation that belongs to the domain and contributes to an understanding of its overall significance. Finally, a subdimension refers to a partial element of a dimension that facilitates its analysis. By using these terms, we aimed to provide a clear, rigorous, and comprehensive framework for conducting HTA.

Table 2 displays the 61 analytical themes in descending order of coding frequency, aligned with the hierarchy derived from the data analysis. Additionally, the table specifies the intervention modalities or dHTs that correspond to each code and lists the studies from which each code originated. The network of relationships among the codes can be found in Multimedia Appendix 5.

**Table 2:** Analytical themes of the thematic analysis presented in descending order of coding frequency and aligned with the hierarchy derived from the data analysis.

Domain (level 1) and dimensions (level 2)			Subdimension (level 3)		Type of dHT^a^
**Description of the technology (n=19, 6.2%)**					Non–face-to-face care models [33,34], mHealth^b^ [36,38-40,55], AI^c^ [41], eHealth [50], digital health [16,17,49,51]
	Credibility and reputation (n=5, 1.6%)	—^d^		mHealth [40,55], digital health [56]	
	Scientific basis (n=5, 1.6%)	—		mHealth [36,39,40], digital health [17,56]	
	Technical evaluation and validation (n=3, 1.0%)	—		mHealth [36], digital health [45]	
	Adoption (n=2, 0.6%)	—		AI [41], digital health [47]	
	Adoption (n=2, 0.6%)	Usage (n=2, 0.6%)		AI [41], digital health [47]	
	Adoption (n=2, 0.6%)	Integration (n=1, 0.3%)		AI [41]	
	Information management (n=2, 0.6%)	—		eHealth [43], digital health [17]	
	Novelty (n=1, 0.7%)	—		Digital health [17]	
**Safety (n=19, 6.2%)**					Non–face-to-face care models [33], mHealth [37-40,52], AI [41], eHealth [43,50], digital health [16,44,49,51,56]
	Clinical safety (n=12, 3.9%)	—		Non–face-to-face care models [33], mHealth [37,38,52], AI [41], eHealth [43,50], digital health [16,44,49,51]	
	Technical safety (n=11, 3.6%)	—		Non–face-to-face care models [33], mHealth [37,39,52], digital health [16,44,49,51,56]	
Clinical efficacy and effectiveness (n=17, 5.5%)					Non–face-to-face care models [33,35], mHealth [38,39,52,53], eHealth [43,50], digital health [16,44,45,47,49,51]
**Economic aspects (n=16, 5.2%)**					Non–face-to-face care models [33-35], mHealth [36,38,39], AI [54], eHealth [42,48,50], digital health [16,45,47,49,51]
	Costs (n=10, 3.2%)	—		Non–face-to-face care models [33,35], mHealth [36,39], AI [54], digital health [16,45,47,49,51]	
	Economic evaluation (n=7, 2.3%)	—		Non–face-to-face care models [33,35], mHealth [38,39], eHealth [42,48], digital health [51]	
	Use of resources (n=4, 1.3%) and efficiency (n=1, 0.3%)	—		Non–face-to-face care models [35], mHealth [36], AI [54], digital health [47,49]	
**Ethical aspects (n=13, 4.2%)**					Non–face-to-face care models [33], mHealth [38,55], AI [41,54], eHealth [42,48,50], digital health [44,46,49,51]
	Equity (n=1, 0.3%)	—		Digital health [19]	
	User control and self-determination (n=1, 0.3%)	—		mHealth [55], digital health [46]	
	Responsibility (n=1, 0.3%)	—		Digital health [44,46]	
	Explainability (n=1, 0.3%)	—		Digital health [46]	
**Human and sociocultural aspects (n=13, 4.2%)**					Non–face-to-face care models [33,35], mHealth [36,38,39], AI [54], eHealth [42,48], digital health [17,49,51]
	User experience (n=7, 2.3%)	—		Non–face-to-face care models [35], mHealth [39,40,52], digital health [17,44,56]	
	Accessibility (n=3, 1.0%)	—		mHealth [52]	
	Acceptability (n=2, 0.6%)	—		mHealth [39], AI [41]	
	Engagement (n=2, 0.6%)	—		Digital health [44,56]	
	Perceived profit (n=1, 0.3%)	—		Digital health [44]	
Organizational aspects (n=3.68%)					Non–face-to-face care models [33], mHealth [38,39], AI [41,54], eHealth [42,43,48,50], digital health [49,51]
**Legal and regulatory aspects (n=10, 3.2%)**					Non–face-to-face care models [33], mHealth [36,38], AI [54], eHealth [42,48,50], digital health [17,49,51]
	Privacy (n=6, 1.9%)	—		mHealth [39,40,52,55], AI [41], digital health [44]	
	Transparency (n=4, 1.3%)	—		mHealth [52,55], AI [41,54]	
	Responsibility (n=1, 0.3%)	—		Digital health [44]	
Description of health problem (n=8, 2.6%)					Non–face-to-face care models [33,34], mHealth [39], eHealth [50], digital health [49,51]
**Content (n=5, 1.6%)**					mHealth [55], eHealth [50], digital health [44,56]
	Information adequacy (n=2, 0.6%)	—		mHealth [55], digital health [56]	
	Intervention adequacy (n=2, 0.6%)	—		Digital health [56]	
**Technical aspects (n=4, 1.3%)**					AI [54], eHealth [42,48], digital health [17]
	Usability (n=10, 3.2%)	—		mHealth [39,52,55], digital health [16,44,45,47,49,56]	
	Adaptability (n=8, 2.6%)	—		Digital health [17]	
	Adaptability (n=8, 2.6%)	Interoperability (n=4, 1.3%)		mHealth [39,52], digital health [16,49]	
	Adaptability (n=8, 2.6%)	Scalability (n=2, 0.6%)		mHealth [52], AI [41]	
	Adaptability (n=8, 2.6%)	Integration of data (n=1, 0.3%)		mHealth [40]	
	Adaptability (n=8, 2.6%%)	Transferability (n=1, 0.3%)		eHealth [48]	
	Quality (n=5, 1.6%)	—		eHealth [43], digital health [17,44,47]	
	Design (n=5, 1.6%)	—		Digital health [56]	
	Design (n=5, 1.6%)	Persuasive design (n=1, 0.3%)		Digital health [56]	
	Technical stability (n=4, 1.3%)	—		mHealth: [38,52], digital health [16,47,49]	
	Aesthetics (n=3, 1.0%)	—		mHealth [39], digital health [44,56]	
	Ease of use (n=3, 1.0%)	—		mHealth [39,40], digital health [56]	
	Accessibility (n=2, 0.6%)	—		mHealth [52], digital health [16]	
	Technical effectiveness (n=1, 0.3%) or performance (n=2, 0.6%)	—		Digital health [17,47]	
	Technical effectiveness (n=1, 0.3%) or performance (n=2, 0.6%)	Reliability (n=6, 1.9%)		mHealth [52,53], digital health [47]	
	Technical effectiveness (n=1, 0.3%) or performance (n=2, 0.6%)	Validity (n=5, 1.6%)		mHealth [52,53], AI [41]	
	Technical effectiveness (n=1, 0.3%) or performance (n=2, 0.6%)	Accuracy (n=2, 0.6%)		Digital health [19]	
	Technical effectiveness (n=1, 0.3%) or performance (n=2, 0.6%)	Sensitivity (n=1, 0.3%)		Digital health [17]	
	Feasibility (n=1, 0.3%)	—		Digital health [47]	
	Generalizability and reproducibility (n=1, 0.3%)	—		AI [54]	
	Interpretability (n=1, 0.3%)	—		AI [54]	
	Customization (n=1, 0.3%)	—		Digital health [44]	
Postmarketing monitoring (n=3, 1%)					mHealth [38], digital health [47]

^a^dHT: digital health technology.

^b^mHealth: mobile health.

^c^AI: artificial intelligence.

^d^N/A: not applicable.

### Research Question 3: Variability of Domains and Dimensions Among Technologies

Our thematic analysis revealed a significant degree of variability and heterogeneity in the number and type of domains and dimensions considered by the methodological frameworks.

In terms of numbers, the variability was quite pronounced when we compared frameworks addressing different types of dHTs. For instance, the thematic analysis of frameworks for assessing telemedicine only identified 9 (75%) domains and 6 (15.8%) dimensions; instead, in frameworks for assessing mHealth, we identified 10 (83.3%) domains, 20 (52.6%) dimensions, and 6 (54.5%) subdimensions, and in frameworks for assessing AI, we identified 8 (66.7%) different domains, 7 (18.4%) different dimensions, and 6 (54.5%) subdimensions.

In terms of the types of domains considered, certain dimensions and domains were identified as more distinctive for one kind of dHT than for another. For instance, clinical efficacy and effectiveness, technical safety, economic evaluation, and user experience were relevant for the evaluation of models of nonpresential health care and mHealth but not for AI. In contrast, there were specific dimensions and domains of mHealth that were not considered in the evaluation of non–face-to-face health care or AI, such as postmarketing monitoring, scientific basis, technical evaluation and validation, user control and self-determination, accessibility, content and adequacy of information, and data interoperability and integration. Finally, specific methodological frameworks for the evaluation of AI included dimensions such as technical aspects, adoption, use, integration, generalizability, reproducibility, and interpretability, which were not considered in the evaluation of telemedicine or mHealth. In conclusion, greater clarity and structuring in the presentation of these ideas are required to facilitate their understanding and assimilation.

### Proposal for Domains, Dimensions, and Subdimensions for dHTA

**Figure 2:**
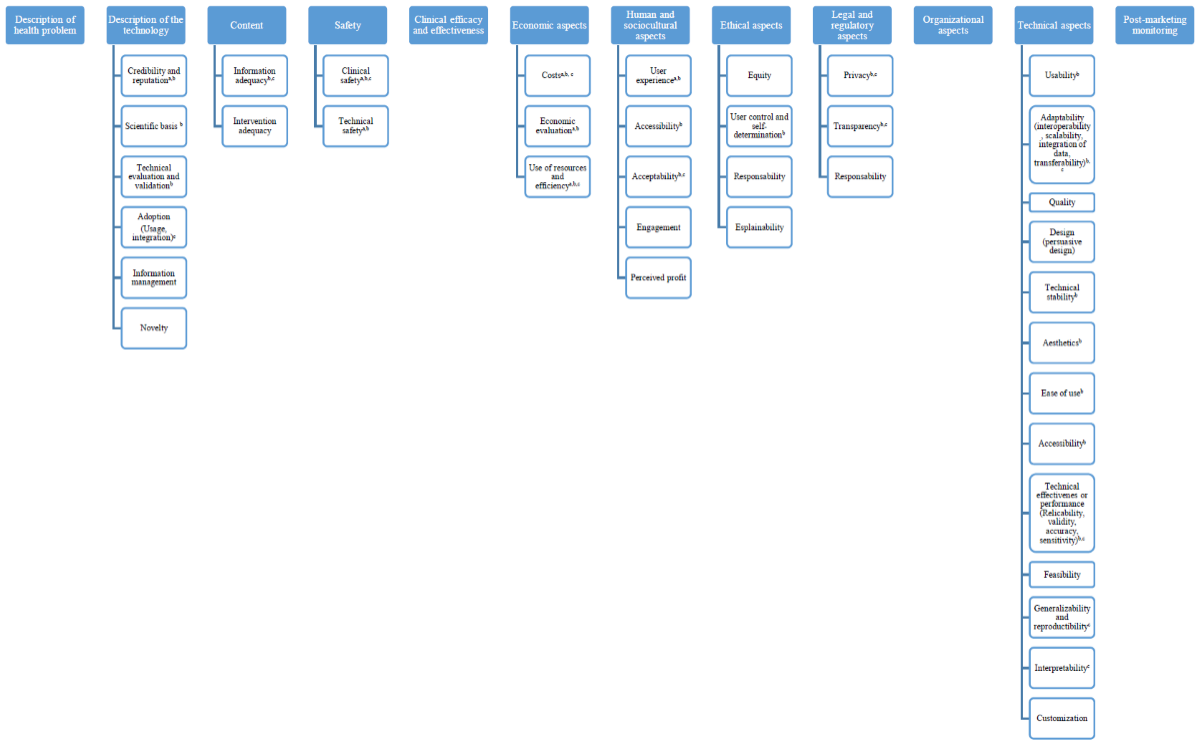
Proposed methodological framework for dHTA. aDimension identified as especially relevant for non–face-to-face care models; bdimension identified as especially relevant for mHealth; cdimension identified as especially relevant for AI; dHTA: digital health technology assessment. A higher-resolution version of this image is available as Multimedia Appendix 6.

These findings led to the development of a proposed methodological framework for dHTA, which comprises domains, dimensions, and subdimensions. These evaluation items were established objectively based on thematically analyzed evidence, without incorporating the researcher’s perspective. Consequently, the proposal for domains, dimensions, and subdimensions emerged from the literature and represents the entirety of identified evaluation domains, dimensions, and subdimensions (n=61). Figure 2 presents a visual representation of the proposed framework comprising 12 domains, 38 dimensions, and their corresponding 11 subdimensions. Notably, the figure highlights certain domains, dimensions, and subdimensions that are particularly relevant to the evaluation of non–face-to-face care models, mHealth, and AI according to the evidence.

## Discussion

### Principal Findings

In recent years, the interest in digital health has increased significantly, giving rise to a myriad of available technologies. This has brought about a profound transformation in health care systems, fundamentally changing the provision and consumption of health care services [9]. However, despite these advancements, the shift toward digital health has been accompanied by challenges. One such challenge is the emergence of a plethora of short-lived implementations and an overwhelming diversity of digital tools, which has created a need for careful evaluation and analysis of the benefits and drawbacks of these technologies [4].

In this context, our ScR aimed to identify the methodological frameworks used worldwide for the assessment of dHTs; determine what domains are considered; and generate, through a thematic analysis, a proposal for a methodological framework based on the most frequently described domains in the literature.

Throughout the ScR, we identified a total of 95 methodological frameworks and tools, of which 19 [16,17,33-47] were directly identified through a systematic search and 75 were indirectly identified through 4 SRs [49-52]. The difference in the number of methodological frameworks identified through the ScR and the 4 evidence synthesis documents [49-52] is attributed to the inclusion of keywords related to the concept of HTA in the search syntax, the exclusion of methodological frameworks published prior to 2011 during the screening process, and the differences in perspectives used for the development of this paper compared to the 4 evidence synthesis documents mentioned earlier. In this sense, these 4 documents [49-52] have analyzed methodological frameworks and tools aimed at evaluating digital health that have not been developed from an HTA perspective despite the authors analyzing them as such. For example, von Huben et al. [51] included in their analysis the Consolidated Standards of Reporting Trials (CONSORT)-EHEALTH tool [97], which aims to describe the information that should be reported in papers and reports that focus on evaluating web- and mHealth-based interventions; Koladas et al [49] included the mobile health evidence reporting and assessment (mERA) checklist [73], which aims to determine the information that should be reported in trials evaluating mHealth solutions; and the European mHealth Hub document [52] includes the Isys Score, which is for cataloguing apps for smartphones.

However, as detailed in the *Results* section, some of the methodological frameworks identified through the ScR were characterized by the authors themselves as being specific for evaluating certain types of dHTs (eg, non–face-to-face care models, mHealth), presenting certain differences according to each typology. It is important to note that the differentiation among various types of dHTs, as described throughout this paper and commonly used in the field of digital health, cannot always be made in a precise and exclusive manner [136]. This is because a technology often can be classified in more than 1 category. For instance, an mHealth solution may use AI algorithms, while simultaneously being integrated into a non–face-to-face care model [137]. In this context, future research should consider using alternative taxonomies or classification methods that are based on the intended purpose of the technology, such as those proposed by NICE in the updated version of the Evidence Standards Framework [18] or the new digital health interventions system classification put forward by WHO [138].

After conducting a thematic analysis of the 26 included studies, we observed that various methodological frameworks include a set of evaluation items, referred to as domains, dimensions, or criteria. These items primarily focus on the safety; effectiveness; technical aspects; economic impact; and ethical, legal, and social consequences of dHTs. However, there is significant heterogeneity among these frameworks in terms of the way they refer to the evaluation items, the quantity and depth of their description, the degree of granularity, and the proposed evaluation methods, especially when comparing frameworks that focus on different types of dHTs. Despite this heterogeneity, most methodological frameworks consider evaluation items related to the 9 domains described by the HTA Core Model of EUnetHTA, while some frameworks propose additional evaluation elements, such as usability [16,44,45,47,49,56], privacy [39-41,44,52,55], and technical stability [16,38,47,49,52] among others. These findings are consistent with earlier research [50,51].

In addition, through the thematic analysis, the heterogeneity identified among the different methodological frameworks included in this ScR was reduced to a total of 61 analytical themes related to various evaluation elements that were arranged in a 3-level vertical hierarchy based on the evidence: level 1 (12 domains), level 2 (38 dimensions), and level 3 (11 subdimensions). At this point, it is pertinent to note that although from the researchers’ perspective, some dimensions could have been classified under different domains (eg, responsibility under ethical aspects) or seen as essential for other kinds of dHTs, an effort was made to maintain the highest degree of objectivity possible. It is for this reason that privacy issues were not described as essential for non–face-to-face care models and why the dimension of accessibility was categorized within the domains of human and sociocultural aspects and technical aspects. This categorization was made because some of the methodological frameworks analyzed associated it with sociocultural elements (eg, evaluating whether users with functional diversity can access the technology and have sufficient ability to use it as expected), while others linked it to technical elements (eg, adequacy of the elements, options, or accessibility functionalities that the system incorporates according to the target audience) [16,52].

The ScR and thematic analysis conducted in this study led to a proposal for a methodological framework for dHTA. This framework was further developed using additional methodologies, such as consensus workshops by the Agency for Health Quality and Assessment of Catalonia (AQuAS), in collaboration with all agencies of RedETS, commissioned by the Ministry of Health of Spain. The final framework is a specific methodological tool for the assessment of dHTs, aimed at describing the domains and dimensions to be considered in dHTA and defining the evidence standards that such technologies must meet based on their associated risk level. The proposed methodological framework enables the assessment of a wide range of dHTs, mainly those classified as medical devices according to the Regulation (EU) 2017/745 for medical devices [139] and Regulation (EU) 2017/746 for in vitro diagnostic medical devices, although it can be adapted to assess dHTs not classified as medical devices [140]. Unlike existing frameworks, it establishes a clear link between the identified domains and dimensions and the evidence standards required for dHTs to meet. This approach will enhance the transparency and consistency of dHTAs and support evidence-based decision-making. The final document was published from November 2023 onward and is available on the RedETS website as well as on the main web page of AQuAS in the Spanish language [141]. From the first week of February, the respective websites have hosted an English version of this document [141], which also is accessible in the INAHTA database. In addition, the Spanish and English versions of the document will be periodically reviewed and, if necessary, adapted to align with emerging technologies and changes in legislation.

### Limitations

Although this ScR was conducted in accordance with the PRISMA-ScR guidelines (Multimedia Appendix 1) and following the recommendations of Peters et al [22] and Pollock et al [23], there were some limitations. First, the search incorporated a block of keywords related to the concept of HTA (see Multimedia Appendix 1) due to the perspective of our ScR, which may have limited the retrieval of some studies to meet the study objective. However, this limitation was compensated for by the analysis of the 3 SRs and the report of the working group on guidelines for the evaluation of mHealth solutions of the European mHealth Hub. Second, much of the literature related to HTA is gray literature and only published on the websites of the authoring agencies. Despite efforts to address this limitation through expert input and a comprehensive search of the websites of the world’s leading agencies, it is possible that certain studies were not identified. Third, the quality and limitations of the analysis conducted by the authors of methodological frameworks and tools included in SRs may have had an impact on the indirect thematic analysis. Therefore, it is possible that some data could have been omitted or not considered during this process. Fourth, the focus on dHTs encompassed within the 3 previously mentioned categories (mHealth, non–face-to-face care models, and medical devices that integrate AI) may have influenced the outcomes of the thematic analysis conducted. Fifth, only methodological frameworks written in Catalan, Spanish, and English were included.

### Comparison With Prior Work

To the best of our knowledge, this is the first ScR to examine the methodological frameworks for dHTA, followed by a thematic analysis with the aim of proposing a new comprehensive framework that incorporates the existing literature in an objective manner and enables the assessment of various technologies included under the concept of digital health. In this sense, existing SRs and other evidence synthesis documents have only analyzed the literature and reported the results in a descriptive manner [36,48,49,51,56,125,126]. Furthermore, this ScR also considered, in addition to scientific literature, gray literature identified by searching the websites of the agencies, thus covering some limitations of previous reviews [50]. Moreover, this review was carried out from the perspective of HTA, addressing a clear need expressed by HTA agencies [16].

Future research should aim to identify what domains and dimensions are relevant at the different stages of the technology life cycle, to establish or develop a standardized set of outcomes for assessing or reporting each domain, and to evaluate the effectiveness and usefulness of the existing methodological frameworks for the different intended users [50,142]. Moreover, future research should aim to determine the specific evaluation criteria that ought to be considered based on the level of risk associated with different types of technologies [51].

### Conclusion

Our ScR revealed a total of 102 methodological frameworks and tools designed for evaluating dHTs, with 19 being directly identified through a systematic search and 83 through 4 evidence synthesis documents. Only 19 of all the identified frameworks were developed from the perspective of HTA. These frameworks vary in assessment items, structure, and specificity, and their proven usefulness in practice is scarce.

The thematic analysis of the 26 studies that met the inclusion criteria led to the identification and definition of 12 domains, 38 dimensions, and 11 subdimensions that should be considered when evaluating dHTs. Building on our results, a methodological framework for dHTA was proposed.

## Appendix

Multimedia Appendix 1 Preferred Reporting Items for Systematic Reviews and Meta-Analysis extension for Scoping Reviews (PRISMA-ScR) checklist [21].

Multimedia Appendix 2 Search strategies for each database.

Multimedia Appendix 3 References excluded at the full-text screening stage.

Multimedia Appendix 4 Methodological frameworks included in systematic reviews.

Multimedia Appendix 5 Network of relationships among the codes.

Multimedia Appendix 6 High-resolution image of Figure 2.

## Abbreviations

AI: artificial intelligence
dHT: digital health technology
dHTA: digital health technology assessment
EUnetHTA: European Network for Health Technology Assessment
HTA: health technology assessment
INAHTA: International Network for Agencies for Health Technology Assessment
mHealth: mobile health
NICE: National Institute for Health and Care Excellence
PICo-D: Population, Phenomenon of interest, Context and Design
PRISMA: Preferred Reporting Items for Systematic Reviews and Meta-Analysis
PRISMA-ScR: Preferred Reporting Items for Systematic Reviews and Meta-Analysis extension for Scoping Reviews
RedETS: Spanish Network of Agencies for Assessing National Health System Technologies and Performance
ScR: scoping review
SNS: Spanish National Health System
SR: systematic review
SNS: Spanish National Health System
WHO: World Health Organization
