# Supplementary material for: Methodological Frameworks and Dimensions to Be Considered in Digital Health Technology Assessment: Scoping Review and Thematic Analysis
Source: J Med Internet Res. 2024 Apr 10;26:e48694. doi: 10.2196/48694 (PMC11043933; doi:10.2196/48694)
Supplement: Multimedia Appendix 2 [file jmir_v26i1e48694_app2.docx]

Search strategies for each database.

| Search Strategy for Medline/ Ovid | | |
| --- | --- | --- |
| # | Search | Results |
| 1 | Mobile Applications/ | 9127 |
| 2 | exp Internet/ | 89348 |
| 3 | exp Cell Phone/ | 19166 |
| 4 | exp Computers, Handheld/ | 10783 |
| 5 | Medical Informatics Applications/ | 2548 |
| 6 | Therapy, Computer-Assisted/ | 6942 |
| 7 | (app or apps).ti,ab. | 27111 |
| 8 | (online or web or internet or digital*).ti. | 90153 |
| 9 | ((online or web or internet or digital*) adj3 (based or application* or intervention* or program* or therap*)).ab. | 49876 |
| 10 | (phone* or telephone* or smartphone* or cellphone* or smartwatch*).ti. | 18803 |
| 11 | ((phone* or telephone* or smartphone* or cellphone* or smartwatch*) adj3 (based or application* or intervention* or program* or therap*)).ab. | 10691 |
| 12 | (mobile health or mhealth or m-health or ehealth or digital health or e-health or emental or e-mental).ti. | 5770 |
| 13 | ((mobile health or mhealth or m-health or ehealth or digital health or e-health or emental or e-mental) adj3 (based or application* or intervention* or program* or therap*)).ab. | 3444 |
| 14 | (mobile* adj3 (based or application* or intervention* or device* or technolog*)).ti,ab. | 11694 |
| 15 | or/1-14 | 241914 |
| 16 | exp Wearable Electronic Devices/ | 15348 |
| 17 | (Wearable* or ((Fitness or Activity) adj3 Track*) or Smartglasses or "Smart Glasses" or (("Head Mounted" or Headmounted or "Head Worn" or "Head Up") adj3 Display*) or "Google Glasses" or SMS or telemed* or telecomm* or "text messag*" or "short message service*" or tablet* or "electronic device*").ab,ti. | 89672 |
| 18 | exp Telemedicine/ | 38067 |
| 19 | exp Monitoring, Physiologic/ | 186664 |
| 20 | exp Machine Learning/ | 36472 |
| 21 | exp Artificial Intelligence/ | 130331 |
| 22 | (Telerehabilitation or Teleradiology or monitoring or "IoT" or "Machine Learning" or "Deep learning" or Telerobotics or Robotics or Robotic or ((Artificial or Computational) and Intelligence)).ab,ti. | 538074 |
| 23 | or/16-22 | 832872 |
| 24 | 15 or 23 | 1035499 |
| 25 | exp Consensus/ | 17148 |
| 26 | Guidelines as Topic/ | 41769 |
| 27 | exp Practice Guidelines as Topic/ | 126187 |
| 28 | Health Planning Guidelines/ | 4145 |
| 29 | guideline.pt. | 16427 |
| 30 | practice guideline.pt. | 28652 |
| 31 | (position statement* or policy statement* or consensus or framework*).ab,ti. | 391828 |
| 32 | (standards or guideline or guidelines or recommendat*).ab,ti. | 653336 |
| 33 | or/25-32 | 1075745 |
| 34 | exp Technology Assessment, Biomedical/ | 11696 |
| 35 | (HTA or ("Health Technology" adj3 (Assessment or Evaluation or Appraisal or Validation))).ab,ti. | 5648 |
| 36 | 34 or 35 | 15009 |
| 37 | 24 and 33 and 36 | 411 |

| Search Strategy for EMBASE | | |
| --- | --- | --- |
| # | Search | Results |
| 1 | 'mobile application'/exp | 18074 |
| 2 | 'internet'/exp | 118773 |
| 3 | 'mobile phone'/exp | 37228 |
| 4 | 'personal digital assistant'/exp | 1679 |
| 5 | 'medical informatics'/exp | 21768 |
| 6 | 'computer assisted therapy'/exp | 15198 |
| 7 | app:ti OR apps:ti | 11355 |
| 8 | online:ti OR web:ti OR internet:ti OR digital*:ti | 145382 |
| 9 | ((online OR web OR internet OR digital*) NEAR/3 (based OR application* OR intervention* OR program* OR therap*)):ab | 88205 |
| 10 | phone*:ti OR telephone*:ti OR smartphone*:ti OR cellphone*:ti OR smartwatch*:ti | 28206 |
| 11 | ((phone* OR telephone* OR smartphone* OR cellphone* OR smartwatch*) NEAR/3 (based OR application* OR intervention* OR program* OR therap*)):ab | 18315 |
| 12 | 'mobile health':ti OR mhealth:ti OR 'm health':ti OR ehealth:ti OR 'digital health':ti OR 'e health':ti OR emental:ti OR 'e mental':ti | 8822 |
| 13 | (('mobile health' OR mhealth OR 'm health' OR ehealth OR 'digital health' OR 'e health' OR emental OR 'e mental') NEAR/3 (based OR application* OR intervention* OR program* OR therap*)):ab | 5650 |
| 14 | (mobile* NEAR/3 (based OR application* OR intervention* OR device* OR technolog*)):ab,ti | 21156 |
| 15 | #1 OR #2 OR #3 OR #4 OR #5 OR #6 OR #7 OR #8 OR #9 OR #10 OR #11 OR #12 OR #13 OR #14 | 386005 |
| 16 | 'wearable computer'/exp | 5844 |
| 17 | wearable*:ab,ti OR (((fitness OR activity) NEAR/3 track*):ab,ti) OR smartglasses:ab,ti OR 'smart glasses':ab,ti OR ((('head mounted' OR headmounted OR 'head worn' OR 'head up') NEAR/3 display*):ab,ti) OR 'google glasses':ab,ti OR sms:ab,ti OR telecomm*:ab,ti OR 'text messag*':ab,ti OR 'short message service*':ab,ti OR tablet*:ab,ti OR 'electronic device*':ab,ti | 150683 |
| 18 | 'telemedicine'/exp | 55509 |
| 19 | 'physiologic monitoring'/exp | 8009 |
| 20 | 'machine learning'/exp | 282070 |
| 21 | 'artificial intelligence'/exp | 54458 |
| 22 | telehealth:ab,ti OR telerehabilitation:ab,ti OR teleradiology:ab,ti OR monitoring:ab,ti OR iot:ab,ti OR 'machine learning':ab,ti OR 'deep learning':ab,ti OR telerobotics:ab,ti OR robotics:ab,ti OR robotic:ab,ti OR (((artificial OR computational) NEAR/3 intelligence):ab,ti) | 936801 |
| 23 | #16 OR #17 OR #18 OR #19 OR #20 OR #21 OR #22 | 1315642 |
| 24 | #15 OR #23 | 1637733 |
| 25 | 'consensus'/de | 81839 |
| 26 | 'health care planning'/exp | 107023 |
| 27 | 'position statement*':ab,ti OR 'policy statement*':ab,ti OR consensus:ab,ti OR framework*:ab,ti | 604301 |
| 28 | standards:ab,ti OR guideline:ab,ti OR guidelines:ab,ti OR recommendations:ab,ti | 1083501 |
| 29 | 'consensus development'/exp | 25509 |
| 30 | #25 OR #26 OR #27 OR #28 OR #29 | 1703662 |
| 31 | 'biomedical technology assessment'/exp | 15586 |
| 32 | hta:ab,ti OR (('health technology' NEAR/3 (assessment OR evaluation OR appraisal OR validation)):ab,ti) | 11451 |
| 33 | #31 OR #32 | 23349 |
| 34 | #25 AND #30 AND #33 | 652 |

| Search Strategy for CINAHL | | |
| --- | --- | --- |
| # | Search | Results |
| 1 | (MH "Mobile Applications") OR (MH "Internet+") OR (MH "Cellular Phone+") OR (MH "Computers, Hand-Held+") OR (MH "Health Informatics+") OR (MH "Therapy, Computer Assisted") | 199,273 |
| 2 | TI ( app OR apps ) OR AB ( app OR apps ) | 11,645 |
| 3 | TI (online OR web OR internet OR digital*) | 70,623 |
| 4 | AB (online OR web OR internet OR digital*) N3 (based OR application* OR intervention* OR program* OR therap*) | 28,933 |
| 5 | TI (phone* OR telephone* OR smartphone* OR cellphone* OR smartwatch*) | 13,095 |
| 6 | AB ((phone* OR telephone* OR smartphone* OR cellphone* OR smartwatch*) N3 (based OR application* OR intervention* OR program* OR therap*)) | 7,670 |
| 7 | TI (mobile health OR mhealth OR m-health OR ehealth OR digital health OR e-health OR emental OR e-mental) | 6,718 |
| 8 | AB ((mobile health OR mhealth OR m-health OR ehealth OR digital health OR e-health OR emental OR e-mental) N3 (based OR application* OR intervention* OR program* OR therap*)) | 2,958 |
| 9 | TI ( (mobile* N3 (based OR application* OR intervention* OR device* OR technolog*)) ) OR AB ( (mobile* N3 (based OR application* OR intervention* OR device* OR technolog*)) ) | 9,208 |
| 10 | S1 OR S2 OR S3 OR S4 OR S5 OR S6 OR S7 OR S8 OR S9 | 270,186 |
| 11 | (MH "Wearable Sensors+") OR (MH "Telemedicine") OR (MH "Telenursing") OR (MH "Monitoring, Physiologic+") OR (MH "Artificial Intelligence+") | 153,309 |
| 12 | TI ( (wearable* OR (((fitness OR activity) N3 track*)) OR smartglasses OR ‘smart glasses’ OR (((‘head mounted’ OR headmounted OR ‘head worn’ OR ‘head up’) N3 display*)) OR ‘google glasses’ OR sms OR telecomm* OR ‘text messag*’ OR ‘short message service*’ OR tablet* OR ‘electronic device*’) ) OR AB ( (wearable* OR (((fitness OR activity) N3 track*)) OR smartglasses OR ‘smart glasses’ OR (((‘head mounted’ OR headmounted OR ‘head worn’ OR ‘head up’) N3 display*)) OR ‘google glasses’ OR sms OR telecomm* OR ‘text messag*’ OR ‘short message service*’ OR tablet* OR ‘electronic device*’) ) | 25,438 |
| 13 | TI ( (telemedicine OR telenursing OR telehealth OR telerehabilitation OR teleradiology OR monitoring OR iot OR ‘machine learning’ OR ‘deep learning’ OR telerobotics OR robotics OR robotic OR (((artificial OR computational) N3 intelligence)) ) OR AB ( (telemedicine OR telenursing OR telehealth OR telerehabilitation OR teleradiology OR monitoring OR iot OR ‘machine learning’ OR ‘deep learning’ OR telerobotics OR robotics OR robotic OR (((artificial OR computational) N3 intelligence)) ) | 150,488 |
| 14 | S11 OR S12 OR S13 | 282,591 |
| 15 | S10 OR S14 | 529,529 |
| 16 | (MH "Consensus") OR (MH "Practice Guidelines") | 87,959 |
| 17 | PT practice guidelines | 15,098 |
| 18 | TI ( (position statement* OR policy statement* OR consensus OR framework*) ) OR AB ( (position statement* OR policy statement* OR consensus OR framework*) ) | 149,779 |
| 19 | TI ( (standards OR guideline OR guidelines OR recommendat*) ) OR AB ( (standards OR guideline OR guidelines OR recommendat*) ) | 500,983 |
| 20 | S16 OR S17 OR S18 OR S19 | 658,326 |
| 21 | TI ( (HTA OR ("Health Technology" N3 (Assessment OR Evaluation OR Appraisal OR Validation))) ) OR AB ( (HTA OR ("Health Technology" N3 (Assessment OR Evaluation OR Appraisal OR Validation))) ) | 3,444 |
| 22 | S15 AND S20 AND S21 | 123 |

| Search Strategy for Cochrane Library | | |
| --- | --- | --- |
| # | Search | Results |
| 1 | MeSH descriptor: [Mobile Applications] explode all trees | 957 |
| 2 | MeSH descriptor: [Internet] explode all trees | 4498 |
| 3 | MeSH descriptor: [Cell Phone] explode all trees | 2103 |
| 4 | MeSH descriptor: [Computers, Handheld] explode all trees | 882 |
| 5 | MeSH descriptor: [Medical Informatics Applications] explode all trees | 9310 |
| 6 | MeSH descriptor: [Therapy, Computer-Assisted] explode all trees | 2435 |
| 7 | ((online or web or internet or digital*)):ti | 13283 |
| 8 | (((online or web or internet or digital*) NEAR/3 (based or application* or intervention* or program* or therap*))):ab | 15367 |
| 9 | ((phone* or telephone* or smartphone* or cellphone* or smartwatch*)):ti | 5910 |
| 10 | (((phone* or telephone* or smartphone* or cellphone* or smartwatch*) NEAR/3 (based or application* or intervention* or program* or therap*))):ab | 7430 |
| 11 | ((mobile health or mhealth or m-health or ehealth or digital health or e-health or emental or e-mental)):ti | 2201 |
| 12 | (((mobile health or mhealth or m-health or ehealth or digital health or e-health or emental or e-mental) NEAR/3 (based or application* or intervention* or program* or therap*))):ab | 22360 |
| 13 | ((mobile* NEAR/3 (based or application* or intervention* or device* or technolog*))):ti,ab,kw | 5755 |
| 14 | ((app or apps)):ti,ab,kw | 6189 |
| 15 | #1 or #2 or #3 or #4 or #5 or #6 or #7 or #8 or #9 or #10 or #11 or #12 or #13 or #14 | 62019 |
| 16 | MeSH descriptor: [Wearable Electronic Devices] explode all trees | 543 |
| 17 | ((Wearable* or ((Fitness or Activity) NEAR/3 Track*) or Smartglasses or "Smart Glasses" or (("Head Mounted" or Headmounted or "Head Worn" or "Head Up") NEAR/3 Display*) or "Google Glasses" or SMS or telemed* or telecomm* or "text messag*" or "short message service*" or tablet* or "electronic device*")):ti,ab,kw | 58602 |
| 18 | MeSH descriptor: [Telemedicine] explode all trees | 3080 |
| 19 | MeSH descriptor: [Monitoring, Physiologic] explode all trees | 12863 |
| 20 | MeSH descriptor: [Machine Learning] explode all trees | 168 |
| 21 | MeSH descriptor: [Artificial Intelligence] explode all trees | 1249 |
| 22 | ((Telerehabilitation or Teleradiology or monitoring or "IoT" or "Machine Learning" or "Deep learning" or Telerobotics or Robotics or Robotic or ((Artificial or Computational) and Intelligence))):ti,ab,kw | 75071 |
| 23 | #16 or #17 or #18 or #19 or #20 or #21 or #22 | 132677 |
| 24 | #15 or #23 | 183380 |
| 25 | MeSH descriptor: [Consensus] explode all trees | 62 |
| 26 | MeSH descriptor: [Guidelines as Topic] this term only | 290 |
| 27 | MeSH descriptor: [Practice Guidelines as Topic] explode all trees | 1670 |
| 28 | MeSH descriptor: [Health Planning Guidelines] explode all trees | 14 |
| 29 | ((position statement* or policy statement* or consensus or framework*)):ti,ab,kw | 19215 |
| 30 | ((standards or guideline or guidelines or recommendat*)):ti,ab,kw | 81678 |
| 31 | #25 or #26 or #27 or #28 or #29 or #30 | 96216 |
| 32 | MeSH descriptor: [Technology Assessment, Biomedical] explode all trees | 183 |
| 33 | ((HTA or ("Health Technology" NEAR/3 (Assessment or Evaluation or Appraisal or Validation)))):ti,ab,kw | 1272 |
| 34 | #32 or #33 | 1369 |
| 35 | #24 and #31 and #34 | 168 |

| Search Strategy for Web of Science (WoS) | | |
| --- | --- | --- |
| # | Search | Results |
| 1 | TS=((app or apps)) | 70402 |
| 2 | TI=((online or web or internet or digital*)) | 552521 |
| 3 | AB=(((online or web or internet or digital*) NEAR/3 (based or application* or intervention* or program* or therap*))) | 210405 |
| 4 | TI=((phone* or telephone* or smartphone* or cellphone* or smartwatch*)) | 63933 |
| 5 | AB=(((phone* or telephone* or smartphone* or cellphone* or smartwatch*) NEAR/3 (based or application* or intervention* or program* or therap*))) | 28484 |
| 6 | TI=(("mobile Health" or mhealth or m-health or ehealth or "digital Health" or e-health or emental or e-mental)) | 12064 |
| 7 | AB=((("mobile Health" or mhealth or m-health or ehealth or "digital Health" or e-health or emental or e-mental) NEAR/3 (based or application* or intervention* or program* or therap*))) | 6087 |
| 8 | TS=((mobile* NEAR/3 (based or application* or intervention* or device* or technolog*))) | 131880 |
| 9 | #8 OR #7 OR #6 OR #5 OR #4 OR #3 OR #2 OR #1 | 924149 |
| 10 | TS=((Wearable* or ((Fitness or Activity) NEAR/3 Track*) or Smartglasses or "Smart Glasses" or (("Head Mounted" or Headmounted or "Head Worn" or "Head Up") NEAR/3 Display*) or "Google Glasses" or SMS or telemed* or telecomm* or "text messag*" or "short message service*" or tablet* or "electronic device*")) | 308213 |
| 11 | TS=((Telerehabilitation or Teleradiology or monitoring or "IoT" or "Machine Learning" or "Deep learning" or Telerobotics or Robotics or Robotic or ((Artificial or Computational) and Intelligence))) | 2178603 |
| 12 | #11 OR #10 | 2439939 |
| 13 | #12 OR #9 | 3240959 |
| 14 | TS=((position statement* or policy statement* or consensus or framework*)) | 1842201 |
| 15 | TS=((standards or guideline or guidelines or recommendat*)) | 3096423 |
| 16 | #14 OR #15 | 4724901 |
| 17 | TS=((HTA or ("Health Technology" NEAR/3 (Assessment or Evaluation or Appraisal or Validation)))) | 7887 |
| 18 | #17 AND #16 AND #13 | 275 |

| Search Strategy for Scopus | | |
| --- | --- | --- |
| # | Search | Results |
| 1 | TITLE-ABS-KEY ( app OR apps ) | 83,265 |
| 2 | TITLE ( online OR web OR internet OR digital* ) | 702,314 |
| 3 | ABS ( ( online OR web OR internet OR digital* ) W/3 ( based OR application* OR intervention* OR program* OR therap* ) ) | 315,115 |
| 4 | TITLE ( ( phone* OR telephone* OR smartphone* OR cellphone* OR smartwatch* ) ) | 72,777 |
| 5 | ABS ( ( phone* OR telephone* OR smartphone* OR cellphone* OR smartwatch* ) W/3 ( based OR application* OR intervention* OR program* OR therap* ) ) | 39,086 |
| 6 | TITLE ( "mobile Health" OR mhealth OR m-health OR ehealth OR "digital Health" OR e-health OR emental OR e-mental ) | 13,84 |
| 7 | ABS ( ( "mobile Health" OR mhealth OR m-health OR ehealth OR "digital Health" OR e-health OR emental OR e-mental ) W/3 ( based OR application* OR intervention* OR program* OR therap* ) ) | 7,964 |
| 8 | TITLE-ABS-KEY ( mobile* W/3 ( based OR application* OR intervention* OR device* OR technolog* ) ) | 203,541 |
| 9 | #1 OR #2 OR #3 OR #4 OR #5 OR #6 OR #7 OR #8 | 1,214,804 |
| 10 | TITLE-ABS-KEY ( wearable* OR ( ( fitness OR activity ) W/3 track* ) OR smartglasses OR "Smart Glasses" OR ( ( "Head Mounted" OR headmounted OR "Head Worn" OR "Head Up" ) W/3 display* ) OR "Google Glasses" OR sms OR telemed* OR telecomm* OR "text messag*" OR "short message service*" OR tablet* OR "electronic device*" ) | 988,338 |
| 11 | TITLE-ABS-KEY ( telerehabilitation OR teleradiology OR monitoring OR "IoT" OR "Machine Learning" OR "Deep learning" OR telerobotics OR robotics OR robotic OR ( ( artificial OR computational ) AND intelligence ) ) | 3,023,224 |
| 12 | #11 OR #12 | 3,906,628 |
| 13 | #12 OR #13 | 4,876,439 |
| 14 | TITLE-ABS-KEY ( "position statement*" OR "policy statement*" OR consensus OR framework* ) | 84,848 |
| 15 | TITLE-ABS-KEY ( standards OR guideline OR guidelines OR recommendat* ) | 4,825,135 |
| 16 | #14 OR #15 | 4,898,830 |
| 17 | TITLE-ABS-KEY ( hta OR ( "Health Technology" W/3 ( assessment OR evaluation OR appraisal OR validation ) ) ) | 9,448 |
| 18 | #9 AND #13 AND #16 | 344 |

| Search Strategy for Tripdatabase | | |
| --- | --- | --- |
| # | Search | Results |
| 1 | (app OR apps OR online OR web OR internet OR digital* OR phone* OR telephone* OR smartphone* OR cellphone* OR smartwatch* OR "mobile Health" OR mhealth OR m-health OR ehealth OR "digital Health" OR e-health OR emental OR e-mental OR Mobile* OR wearable OR Display OR smartglasses OR "Smart Glasses" OR "Google Glasses" OR sms OR telemed* OR telecomm* OR "text messag*" OR "short message service*" OR tablet* OR "electronic device*" OR telerehabilitation OR teleradiology OR monitoring OR "IoT" OR "Machine Learning" OR "Deep learning" OR telerobotics OR robotics OR robotic OR "Artificial Intelligence") AND (hta OR ( "health technology" AND ( assessment OR evaluation OR appraisal OR validation ) )) | 801 |
