# Supplementary material for: Methodological Frameworks and Dimensions to Be Considered in Digital Health Technology Assessment: Scoping Review and Thematic Analysis
Source: J Med Internet Res. 2024 Apr 10;26:e48694. doi: 10.2196/48694 (PMC11043933; doi:10.2196/48694)
Supplement: Multimedia Appendix 3 [file jmir_v26i1e48694_app3.docx]

References excluded at the full-text screening stage.

| Year | Author | Title | Reason of exclusion |
| --- | --- | --- | --- |
| 2011 | Eisenstein, E. L. | Defining a framework for health information technology evaluation | Phenomenon of Interest |
| 2011 | Golterman, L. | Evaluating web sites: reliable child health resources for parents | Phenomenon of Interest |
| 2013 | Gizbert, J. | NICE embracing digital technology | Phenomenon of Interest |
| 2015 | Waterman, J. | A framework for coverage decisions for digital health technologies | Phenomenon of Interest |
| 2016 | Arnold, K. | Principles for the evaluation of telemedicine applications: Results of a systematic review and consensus process | Language |
| 2016 | Craven, C. K. | Evidence-based Health Informatics Frameworks for Applied Use | Phenomenon of Interest |
| 2016 | Ekeland, A. G. | Assessment of mast in european patient-centered telemedicine pilots | Phenomenon of Interest |
| 2016 | Frosini, F. | Integrated HTA-FMEA/FMECA methodology for the evaluation of robotic system in urology and general surgery | Phenomenon of Interest |
| 2016 | Garell, C. | A legal framework to support development and assessment of digital health services | Phenomenon of Interest |
| 2016 | Lau, F. | Handbook of eHealth evaluation: an evidence-based approach | Phenomenon of Interest |
| 2016 | Lee, T. T. | Evaluation of Health Information Technology - Key Elements in the Framework | Phenomenon of Interest |
| 2016 | Leigh, S. | Comparing applets and oranges: barriers to evidence-based practice for app-based psychological interventions | Phenomenon of Interest |
| 2016 | McName, P. | Designing and undertaking a health economics study of digital health interventions | Phenomenon of Interest |
| 2017 | Gómez, S. | Assessing mhealth: Proposal of a new framework | Publication Type |
| 2017 | Hofmann, B. | Toward a Method for Exposing and Elucidating Ethical Issues with Human Cognitive Enhancement Technologies | Phenomenon of Interest |
| 2017 | Hostgaard, A. | Constructive eHealth evaluation: lessons from evaluation of EHR development in 4 Danish hospitals | Phenomenon of Interest |
| 2018 | Barna, A. | Evidence required by Health Technology Assessment and Reimbursement bodies evaluating diagnòstic or prognostic algorithms that include omics data | Phenomenon of Interest |
| 2018 | Greaves, F. | What is an appropriate level of evidence for a digital health intervention? | Publication Type |
| 2018 | Kloc, K. | How regulatory paths for e-Health solutions could further impact market access in the United States and the European Union. | Publication Type |
| 2018 | Lee, T. T. | US Food and Drug Administration precertification pilot program for digital health software: weighing the benefits and risks | Phenomenon of Interest |
| 2018 | Mesana, L. | Evaluating digital Health technologies: A review of HTA guidance and assessments in Europe. | Publication Type |
| 2018 | Ridley, C. | Health technology assessment of Mobile Health Applications: slow progress in a fast-paced industry? | Publication Type |
| 2019 | Biggs, J. S. | Digital health benefits evaluation frameworks: building the evidence to support Australia's National Digital Health Strategy | Phenomenon of Interest |
| 2019 | Cortez, N. | Digital health and regulatory experimentation at the FDA | Phenomenon of Interest |
| 2019 | HIQA | Guide to a review programme of eHealth services in Ireland | Phenomenon of Interest |
| 2019 | Kloc, K. | Assessment of digital Health techonologies – Comparison of evidence frameworks of NICE and HAS. | Publication Type |
| 2019 | Moshi, M. | mHealth app evaluation framework for reimbursement decision-making | Publication Type |
| 2019 | Moshi, M. | Evaluation of Mobile Health Applications: Is Regulatory Policy Up to the Challenge? | Phenomenon of Interest |
| 2019 | Puigdomenech, E. | Development of an mHhealth interventions evaluation tool. What has been done and next steps | Publiation Type |
| 2019 | Slack, D. | Assessing the local interpretability of machine learning models | Phenomenon of Interest |
| 2019 | Taakács, B. | Setting the scope for assessing e-health technologies in Hungary | Publication Type |
| 2019 | Thadaney Israni, S | Artificial intelligence in health care: The Hope, the hype, the promise, the peril | Phenomenon of Interest |
| 2020 | Arcaa, E. | PMU57 Where Are WE Headed with Digital Therapeutics? a Systematic Review of the Evidence | Publication Type |
| 2020 | Francis, L. | PMH37 Bridging the Gap Between HTA and Digital Health Technologies in England - What Are the Challenges Faced by NICE in Assessing Psychological Therapies? | Phenomenon of Interest |
| 2020 | Garcia­Saez, G. | Next generation health technology assessment to support patient-centred, societally oriented, real-time decision-making in diabetes | Publication Type |
| 2020 | Godfrey, A. | BioMeT and algorithm challenges: a proposed digital standardized evaluation framework | Phenomenon of Interest |
| 2020 | Inal, Y. | Usability evaluations of mobile mental health technologies: Systematic review | Phenomenon of Interest |
| 2020 | Liaw, S. T. | Evaluation of Digital Health & Information Technology in Primary Care | Purpose |
| 2020 | Ni, M. | The Lean and Agile Multi-dimensional Process (LAMP) a new framework for rapid and iterative evidence generation to support health-care technology design and development | Publication Type |
| 2020 | Powell, A. | A Patient-Centered Framework for Measuring the Economic Value of the Clinical Benefits of Digital Health Apps: Theoretical Modeling | Phenomenon of Interest |
| 2020 | Tomashchuk, O. | Threat and Risk Management Framework for eHealth IoT Applications | Phenomenon of Interest |
| 2020 | Yang, Y. | Intervention and Evaluation of Mobile Health Technologies in Management of Patients Under-going Chronic Dialysis: Scoping Review | Phenomenon of Interest |
| 2021 | Forsyth, J. R. | Application of the National Institute for Health and Care Excellence evidence standards framework for digital health technologies in assessing mobile | Phenomenon of Interest |
| 2021 | Hendrix, N. | Assessing the Economic Value of Clinical Artificial Intelligence: Challenges and Opportunities | Phenomenon of Interest |
| 2021 | Homma, K. | Developing a Framework for Evaluating Robotic Care Devices in the Introduction Phase | Phenomenon of Interest |
| 2021 | Tarricone, R. | Distinguishing features in the assessment of mHealth apps | Phenomenon of Interest |
| 2021 | Unsworth, H. | The NICE Evidence Standards Framework for digital health and care technologies â€“ Developing and maintaining an innovative evidence framework with global impact | Phenomenon of Interest |
| 2021 | Vervoort, D. | Health Technology Assessment for Cardiovascular Digital Health Technologies and Artificial Intelligence: Why is it Different? | Publication Type |
| 2021 | Jeindl, R. | Technology assessment of digital health applications for reimbursement decisions | Language |
