# Supplementary material for: Methodological Frameworks and Dimensions to Be Considered in Digital Health Technology Assessment: Scoping Review and Thematic Analysis
Source: J Med Internet Res. 2024 Apr 10;26:e48694. doi: 10.2196/48694 (PMC11043933; doi:10.2196/48694)
Supplement: Multimedia Appendix 4 [file jmir_v26i1e48694_app4.docx]

## Methodological frameworks included in systematic reviews.

| Evidence Synthesis document, year | Methodological framework included in SRS, year | Country | Affiliation | Assessment specificity | Assessment Type | Number of domains | Domains |  |
| --- | --- | --- | --- | --- | --- | --- | --- | --- |
| Methodological frameworks focused on the evaluation of remote care models | | | | | | | | |
| Kolasa and Kozinski (2020) [49] | A Proposed Framework for Economic Evaluation of Telemedicine, 2009 [68] | USA | Investigation Group | Specific assessment: economic aspects | NA | 1 | Economic evaluation* |  |
| Kolasa and Kozinski (2020) [49] | An Assessment Framework for Telemedicine Applications, 1999 [65] | Canada | Investigation Group | Overall evaluation | By domains | 10 | Context; time elements; cost elements; security; quality; effectiveness; effectiveness; cost comparison; cost-effectiveness; acceptability; access |  |
| Vis et al (2020) [50] | Assessment of telemedicine applications, 2021 [66] | Finland | Investigation Group | Overall evaluation | By domains | eleven | Assessment of technical aspects (image/voice technical quality, reliability, validity and other characteristics); efficacy (diagnostic quality, quality of life, clinic, management process, know-how and non-health outcomes of the patient); user evaluation (quality, usability and satisfaction with the technology); costs (investment, user charge for equipment, communication lines, staff salaries and education costs); other relevant costs (housing, administration, etc.); patient costs; patient lost work hours/time off; intangible costs related to health); study design (randomization, before/after comparison, and control groups); economic evaluation methods (costs, cost-effectiveness, cost-benefit ratio and cost-utility analysis); sensitivity analysis |  |
| von Huben et al (2021) [51] | Beintner, 2019 [59] | Germany | Investigation Group | Overall evaluation | NA | NA | Address adherence in each publication regarding online interventions; Please provide details on how adherence was implemented and how usage metrics were derived in the methods section; Include information about adherence in your abstract; Please provide detailed information on adherence in the results section and in the flowchart of the Consolidated Test Reporting Standards; address the potential impact of adherence on intervention outcomes in the discussion section. |  |
| Vis et al (2020) [50] | Clinical Value Compass, 2012 [69] | Israel | Investigation Group | Overall evaluation | By domains | 4 | Health related to quality of life (physical aspects and emotional aspects); patient satisfaction (modality and technological aspects); costs (admissions to the emergency room and hospitalizations); clinical status (relapsed disability, severity of symptoms). |  |
| Vis et al (2020) [50] | Comprehensive Telemedicine Evaluation, 2007 Model [57] | Venezuela | Investigation Group | Overall evaluation | business modeling | 4 | Quality; accessibility; costs; acceptability |  |
| Vis et al (2020) [50] | Health Services Research framework (HSR), 2005 [64] | USA | Investigation Group | Overall evaluation | By domains | 3 | Accessibility and timeliness of care; care costs; quality of care (structure: speed and technical quality of transmission, adequacy of equipment, skills, costs and accessibility; process: sensitivity and specificity of the diagnosis and evidence base of the treatment plan; outcome: short term: clinical results; intermediate : adherence, acceptability and satisfaction; long term: quality of life, health or functional status) |  |
| Vis et al (2020) [50] | Innovating in Healthcare Framework, 2017 [70] | Netherlands | Investigation Group | Overall evaluation | business modeling | 8 | Understanding the black box; depth of investigation; secondary risks; financial considerations; regulatory aspects; potential competition from other technologies; probability of obtaining a patent; production considerations |  |
| Vis et al (2020) [50] | Multi-method telemedicine application evaluation, 2006 [60] | Australia | Investigation Group | Overall evaluation | By domains | 5 | Utilization component; clinical component; organizational component; technical component; cost component |  |
| von Huben et al (2021) [51] | Rajan, 2019 [67] | USA | Investigation Group | Overall evaluation | NA | 2† | costs; economic aspects* |  |
| von Huben et al (2021) [51] | Rojahn, 2016 [72] | United States and United Kingdom | Investigation Group | Overall evaluation | NA | 2† | costs; organizational aspects* |  |
| Vis et al (2020) [50] | Staged approach to evaluation of telemedicine, 1996 [61] | USA | Investigation Group | Overall evaluation | by phases | 4 | Technical efficiency (accuracy and reliability); specific objectives of the System (evaluation of the overall impact on access, quality and cost); system analysis (evaluation of the overall impact on access, quality and cost for the system); external validity (evaluation of the overall impact on access, quality and cost in different systems) |  |
| Vis et al (2020) [50] | Telehealth Evaluation Framework, 2001 [71] | Canada | Investigation Group | Overall evaluation | By domains | 4 | Individual structure (patient access to services, patient acceptability, provider training, and provider change in practice); organizational structure (scheduling, suitability of team location, culture, cost, and team effectiveness); care process (satisfaction, effectiveness of the interaction, and management of the care process); individual outcomes (satisfaction, quality of life, functional status, number of readmissions, and adverse effects); organizational results (use of resources, profitability and utilization) |  |
| Vis et al (2020) [50] | Telemedicine Evaluation Plan, 2001 [127] | Norway | Investigation Group | Overall evaluation | By domains | 9 | Assessment of the expectations and reactions of professional users; customer reactions; logistics, organization and technology; medical capital gains; patient waiting time; movement of patients; economy; transfer of powers; recruitment/retention of staff |  |
| Vis et al (2020) [50] | Telemedicine Quality Control System (TM-QC), 2007 [62,63] | Italy | Investigation Group | Overall evaluation | Hybrid | 5 | Phase 1: preliminary evaluation of characteristics; classification (application area and users); preliminary evaluation and areas for improvement; Phase 2: technical file detailing the performance and functionalities; a quality assessment checklist (product requirements related to patient safety, data privacy and integrity, transmission security, system requirements, product support, software certification, the standards used, documentation, medico-legal implications and legal validity, effectiveness of the telemedicine health service and communication with users, design, manufacture and testing of the product, and evaluation of economic and social aspects) |  |
| Vis et al (2020) [50] | Three-dimensional model for telemedicine evaluation, 2005 [58] | USA | Investigation Group | Overall evaluation | By domains | 5 | Security; effectiveness; access; quality and costs |  |
| Methodological frameworks focused on the evaluation of mHealth technologies | | | | | | | | |
| European mHealth Hub, (2020) [125], Vis et al (2020) [50], Kolasa and Kozinski (2020) [49], von Huben, A. (2021) [51] | Andalusian mHealth Strategy, 2012 [74] | Spain | Government organization | Overall evaluation | By domains | 4 | Design and suitability (suitability, accessibility, design, usability); information quality and security (suitability for audience, transparency, authorship, update/revision information, content and information resources, risk management); provision of services (technical support, e-commerce, broadband, advertising); confidentiality and privacy (data protection and privacy, logical security) |  |
| European mHealth Hub (2020) [125] | App check [95] | Germany | Government organization | Overall evaluation | NA | 8† | Privacy; transparency; security; technical stability; efficacy, effectiveness and efficiency; accessibility; user experience and usability; protection* |  |
| Kolasa and Kozinski (2020) [49] | App Evaluation Model – American Psychiatric Association, 2022 [75] | USA | Non Governmental Organization | Overall evaluation | by phases | 5 | Context and access; Privacy & Security; clinical basis; usability; integration of data towards the therapeutic objective |  |
| European mHealth Hub (2020) [125] | AppKRI | Germany | Government organization | Overall evaluation | NA | 12† | Privacy; transparency; security; reliability; validity; interoperability; technical stability; efficacy, effectiveness and efficiency; accessibility; scalability; user experience and usability; protection* |  |
| European mHealth Hub (2020) [125] | AppQ [77] | Germany | Government organization | Overall evaluation | NA | 12† | Privacy; transparency; security; reliability; precision; interoperability; technical stability; efficacy, effectiveness and efficiency; accessibility; scalability; user experience and usability* |  |
| European mHealth Hub (2020) [125] | BfArM DiGA Fast Track and Guidance Document [82] | Germany | Government organization | Overall evaluation | NA | 10† | Privacy; transparency; security; validity; interoperability; technical stability; efficacy, effectiveness, efficiency; accessibility; user experience and usability; protection* |  |
| European mHealth Hub (2020) [125] | CEN-ISO/DTS 82304-2 “Health and wellness apps - Quality and reliability criteria across the life cycle” [88] | Global | Non Governmental Organization | Overall evaluation | NA | 12† | Privacy; transparency; security; reliability; validity; interoperability; technical stability; efficacy, effectiveness and efficiency; accessibility; scalability; user experience and usability; protection* |  |
| European mHealth Hub (2020) [125] | Continua Design Guidelines (CDG) [89] | International | Non Governmental Organization | Overall evaluation | NA | 9† | Privacy; transparency; security; reliability; interoperability; technical stability; efficacy, effectiveness and efficiency; scalability; security* |  |
| European mHealth Hub (2020) [125] | HL7 Consumer Mobile Health Application Functional Framework (cMHAFF) [128] | International | Non Governmental Organization | Overall evaluation | NA | 9† | Privacy; transparency; security; interoperability; technical stability; efficacy, effectiveness and efficiency; accessibility; user experience and usability; protection* |  |
| European mHealth Hub (2020) [125] | Criteria catalogue for self-declaration of the quality of health apps [129] | Swiss | Government organization | Overall evaluation | NA | 8† | Privacy; transparency; security; validity; interoperability; technical stability; efficacy, effectiveness and efficiency; protection* |  |
| European mHealth Hub (2020) [125] | DEKRA Certification - MEDAPPCARE | France | Non Governmental Organization | Overall evaluation | NA | 6† | Privacy; transparency; security; technical stability; efficacy, effectiveness and efficiency; protection* |  |
| von Huben et al (2021) [51] | Dick, 2020 [81] | Ireland, UK and Malawi | Investigation Group | Specific evaluation: clinical effectiveness | NA | 1† | Clinical effectiveness* |  |
| von Huben et al (2021) [51] | EU Draft Consard Ltd, 2016 [91] | Unknown | Government organization | Overall evaluation | By domains | 9 | desirability; credibility; security; protection; transparency; usability; effectiveness; stability; reliability |  |
| European mHealth Hub (2020) [125] | GDD AppSotre [76] | Netherlands | Government organization | Overall evaluation | NA | 10† | Privacy; transparency; reliability; validity; technical stability; efficacy, effectiveness and efficiency; accessibility; user experience and usability; protection* |  |
| von Huben et al (2021) [51] | Gorski, 2016 [83] | USA | Investigation Group | Specific evaluation: value proposition | NA | NA | NA |  |
| Kolasa and Kozinski (2020) [49] | Guidelines for Reporting of Health Interventions Using Mobile Phones: Mobile Health (mHealth) Evidence Reporeting and Assessment (mERA) Checklist [73] | Unknown | Government organization | Overall evaluation | NA | 16 | Infrastructure (population level); Technological platform; interoperability/context of health information systems; intervention; usability/ content testing; user feedback; access of individual participants; cost evaluation; adoption tickets/program tickets; limitations for scalability; context adaptability; replicability; data security; compliance with national guidelines and regulatory statutes; intervention fidelity |  |
| von Huben et al (2021) [51] | Hogaboam, 2018 [84] | USA | Investigation Group | Overall evaluation | Hybrid | 5 | Financial perspective (acquisition costs, economies of scale, maintenance cost, decrease in clinical or hospitalization costs); technical perspective (reliability, ease of use, impact on productivity, interoperability, ease of access to data); organizational perspective (necessary training, technical support, data support, competitive advantage); patients perspective (patient experience, clinical benefits for patients, privacy and security) |  |
| von Huben et al (2021) [51] | Huckvale, 2019 [85] | Australia | Investigation Group | Specific assessment: privacy | NA | 1† | Privacy* |  |
| von Huben et al (2021) [51] | IRBs advisor, 2017 [94] | USA | Non Governmental Organization | Specific evaluation: ethical aspects | NA | 2† | Ethical aspects* |  |
| European mHealth Hub (2020) [125] | Isys Score | Spain | Non Governmental Organization | Overall evaluation | NA | 4† | Privacy; security; validity; efficacy, effectiveness and efficiency* |  |
| von Huben et al (2021) [51] | Maar, 2017 [86] | Canada | Investigation Group | Specific evaluation: effectiveness | By domains | 4 | The participating patients comprise the main target population of the intervention, in this case, people with hypertension are enrolled in the study; participating providers are providers of care and services whose work is altered to some degree by the intervention; Community and organization members are people whose immediate social environment impacts the intervention, for example, those who enable implementation or approve workflow changes. They can also be decision makers; members of the health system and environment are people or structures that influence implementation at the systems level, such as local and district level decision makers and national health policy makers |  |
| von Huben et al (2021) [51] | McMillan, 2016 [87] | United Kingdom | Investigation Group | Overall evaluation | By domains | 9 | Purpose of the app; Planning and development; usability; initial evaluation and adaptation; cognitive-behavioral techniques; behavior maintenance and relapse prevention; assessment; documentation; Data Protection |  |
| Kolasa and Kozinski (2020) [49] | mHealth Assessment Conceptualization of a Global Framework, 2017 [78] | Norway and Spain | Investigation Group | Overall evaluation | By domains | 4 | Technical maturity; risks; benefits; necessary resources |  |
| European mHealth Hub (2020) [125] | mHealthBelgium, 2022 [130] | Belgium | Government organization | Overall evaluation | Hybrid | 3† | Privacy; transparency; interoperability; efficacy, effectiveness and efficiency; accessibility; scalability; user experience and usability; protection* |  |
| European mHealth Hub (2020) [125] | MindsApps.dk [80] | Denmark | Government organization | Overall evaluation | phases | 5† | Privacy; security; reliability; user experience and usability; protection* |  |
| von Huben et al (2021) [51] | Mobile Medical Application Evaluation Module, 2020 [126] | Australia | Investigation Group | Overall evaluation | By domains | 10 | Description and technical characteristics; current use of technology; effectiveness; security; efectivity cost; organizational aspects; ethical aspects; legal aspects; post-market monitoring; social aspects |  |
| von Huben et al (2021) [51] | Mookherji, 2015 [131] | Switzerland and USA | Investigation Group | Specific evaluation: clinical effectiveness | NA | 1† | Clinical effectiveness* |  |
| European mHealth Hub (2020) [125] | My Health Apps | United Kingdom | Non Governmental Organization | Overall evaluation | NA | 6† | Privacy; transparency; security; interoperability; efficacy, effectiveness and efficiency; user experience and usability* |  |
| European mHealth Hub (2020) [125] | MySNS Selecção [132] | Portugal | Government organization | Overall evaluation | by phases | 9† | Privacy; transparency; security; reliability; validity; technical stability; efficacy, effectiveness and efficiency; accessibility; protection* |  |
| von Huben et al (2021) [51] | Nielsen [133] | Ireland | Non Governmental Organization | Overall evaluation | NA | 7† | Privacy and data protection; evidence-based information; functionality; security and authentication; usability and user experience; effectiveness and impact; interoperability* |  |
| European mHealth Hub (2020) [125] | ORCHA Review process [134] | United Kingdom | Non Governmental Organization | Overall evaluation | NA | 12 | Privacy; transparency; security; reliability; validity; interoperability; technical stability; efficacy, effectiveness and efficiency; accessibility; scalability; user experience and usability; protection* |  |
| European mHealth Hub (2020) [125] | Our Mobile Health | United Kingdom | Non Governmental Organization | Overall evaluation | NA | 8 | Privacy; security; interoperability; technical stability; efficacy, effectiveness and efficiency; accessibility; user experience and usability; protection* |  |
| European mHealth Hub (2020) [125] | PAS 277:2015 Health and wellness apps – Quality criteria across the life cycle – Code of practice [79] | United Kingdom | Government organization | Overall evaluation | NA | eleven | Privacy; transparency; security; reliability; interoperability; technical stability; efficacy, effectiveness and efficiency; accessibility; scalability; user experience and usability; security* |  |
| von Huben et al (2021) [51] | Philpott, 2017 [90] | USA | Investigation Group | Overall evaluation | by phases | NA | Clinical effectiveness* |  |
| European mHealth Hub (2020) [125], Kolasa and Kozinski (2020) [49] | Rerport of the Working Group on mHealth Assessment Guideliness, 2021 [52] | Unknown | Government organization | Overall evaluation | By domains | 5† | Privacy; transparency; reliability; validity; interoperability* |  |
| European mHealth Hub (2020) [125] | Servicio de certificación de aplicaciones de la Fundación Tic Salut y Social (FTTS) [135] | Spain | Government organization | Overall evaluation | Hybrid | 4† | Privacy; transparency; security; reliability; technical stability; effectiveness, efficacy and efficiency; accessibility; user experience and usability and security* |  |
| von Huben et al (2021) [51] | Sax, 2018 [92] | Netherlands | Investigation Group | Specific evaluation: ethical and legal aspects | By domains | 2 | ethical analysis; legal analysis |  |
| von Huben et al (2021) [51] | Wyatt, 2018 [93] | United Kingdom | Investigation Group | Specific evaluation: clinical effectiveness | NA | 1† | Clinical effectiveness* |  |
| Methodological frameworks focused on the evaluation of solutions based on AI | | | | | | |  | |
| - | - | - | - | - | - | - | - |  |
| Methodological frameworks focused on the evaluation of eHealth technologies | | | | | | | | |
| von Huben et al (2021) [51] | Bergmo, 2015 [96] | Norway | Investigation Group | Specific assessment: economic aspects | NA | 3† | Economic aspects (cost-benefit, cost-effectiveness, cost-utility) |  |
| von Huben et al (2021) [51] | CONSORT eHealth, 2011 [97] | Canada | Investigation Group | Overall evaluation | NA | 5† | Health problem and current use of technology; description and technical characteristics of the technology; security; clinical effectiveness; ethical aspects* |  |
| Vis et al (2020) [50] | CHEATS, 2002 [98,99] | United Kingdom | Investigation Group | Overall evaluation | By domains | 6 | Cynical aspects; human and organizational aspects; educational aspects; administrative aspects; technical aspects; social aspects |  |
| Vis et al (2020) [50] | Device Selection Matrix , 2005 [100] | Unknown | NA | Overall evaluation | by phases | NA | usability; robustness; unit size; ease of configuration; costs; weight; availability |  |
| Vis et al (2020) [50] | Eight-dimension sociotechnical modelo of safe and effective IT use, 2013 [101] | India | Investigation Group | Overall evaluation | By domains | 8 | Software and machinery; content; user interface; staff; organizational features; State and federal regulations; workflows and communication; monitoring |  |
| European mHealth Hub (2020) [125] | Good Practice Guidelines on Health Apps and Smart Devices (Mobile Health or mHealth) [102] | France | Government organization | Overall evaluation | NA | 12† | Privacy; transparency; security; reliability; validity; interoperability; technical stability; efficacy, effectiveness and efficiency; accessibility; scalability; user experience and usability; security* |  |
| von Huben et al (2021) [51] | Health Information and Quality Authority (IE) [103] | Ireland | Government organization | Specific assessment: legal aspects | NA | NA | NA |  |
| von Huben et al (2021) [51] | Jurkeviciute, 2018 [104] | Sweden | Investigation Group | Overall evaluation | NA | 1† | Clinical effectiveness* |  |
| Vis et al (2020) [50] | Stepped evaluation of eHealth services, 2013 [105] | Sweden | Investigation Group | Overall evaluation | by phases | NA | Phase 1: identify and classify goals and effects; phase 2: determine requirements, needs and preconditions (technical, operational and financial); phase 3: economic impact; phase 4: implementation strategies; phase 5: rewards and incentives; Phase 6: Get a full picture of the service. |  |
| Vis et al (2020) [50] | Tehcnology, Economic, Market, Political, Evaluation, Social and Transofrmation (TEMPEST), 2012 [106] | United Kingdom | Investigation Group | Overall evaluation | By domains | 7 | Technology; economic aspects; market; legal aspects; assessment; social aspects; transformation |  |
| Vis et al (2020) [50] | Unified eValuation using Ontology (UVON), 2016 [107] | Sweden and Switzerland | Investigation Group | Overall evaluation | By domains | 3 | Effectiveness; security; empowerment |  |
| Methodological frameworks focused on the evaluation of digital health technologies | | | | | | | | |
| von Huben et al (2021) [51] | Academy of Medical Sciences (UK) [108] | United Kingdom | Non Governmental Organization | Overall evaluation | By domains | 2 | Security; transparency |  |
| von Huben et al (2021) [51] | Australian Commission on Safety and Quality in Health Care, 2020 [109] | Australia | Government organization | Overall evaluation | NA | 7 | Description and technical characteristics of the technology; security; clinical effectiveness; ethical analysis; organizational aspects; social and patient aspects; legal aspects |  |
| von Huben et al (2021) [51] | Department of Health & Social Care (UK), 2021 [110] | United Kingdom | Government organization | Overall evaluation | by principles | 12 | How to operate ethically; have a clear value proposition; usability and accessibility; technical guarantee; Data Protection; data transparency; cybersecurity; regulation; interoperability and open standards; generate evidence regarding clinical, social, economic, and behavioral benefits; define a business strategy |  |
| von Huben et al (2021) [51] | Drury, 2018 [111] | Philippines | Non Governmental Organization | Overall evaluation | NA | 4† | Health problem and current use of technology; description and technical characteristics of the technology; costs and economic evaluation; organizational aspects* |  |
| von Huben et al (2021) [51] | European Commission. Synopsis report, consultation:Transformation health and care in the digital single market, 2018 [112] | Unknown | Government organization | Overall evaluation | NA | 7† | Description and technical characteristics of the technology; security; costs and economic aspects; social and patient aspects; legal aspects* |  |
| European mHealth Hub, (2020) [125], Kolasa and Kozinski (2020) [49], von Huben, A. (2021) [51] | Evidence Standards Framework for Digital Health Technologies, 2018 [19] | United Kingdom | Government organization | Overall evaluation | By domains | 9† | Health problem and current use of technology; description and technical characteristics of the technology; security; clinical effectiveness; costs and economic aspects; social and patient aspects; legal aspects* |  |
| von Huben et al (2021) [51] | Federal Ministry of Health, 2020 [113] | Germany | Government organization | Overall evaluation | NA | 6† | Health problem and current use of technology; description and characteristics of the technology; security; clinical effectiveness; organizational aspects; legal aspects* |  |
| von Huben et al (2021) [51] | Guide to the specific features of clinical evaluation of a connected medical device (CMD) in view of its application for reimbursement, 2019. [114] | France | Government organization | Overall evaluation | NA | 6† | Health problem and current use of technology; description and technical characteristics of the technology; security; clinical effectiveness; costs and economic evaluation; organizational aspects* |  |
| European mHealth Hub, (2020) [125], Kolasa and Kozinski (2020) [49], von Huben, A. (2021) [51] | How we Assess Health Apps and Digital Tools – NHS Digital Guidelines [115] | United Kingdom | Government organization | Overall evaluation | NA | 4† | Health problem and current use of technology; description and technical characteristics of the technology; security; clinical effectiveness* |  |
| von Huben et al (2021) [51] | Lennon, (2017) [116] | United Kingdom | Investigation Group | Specific assessment: organizational aspects | By domains | 1 | Organizational aspects (interoperability, risk, clinical improvement, national regulation, infrastructure, market incoherence, ICT infrastructure, organizational culture and discontinuity, resources, workload and professional trust, training, access to digital resources, digital literacy and access, lifestyle, security and confidence) |  |
| von Huben et al (2021) [51] | McNamee, 2016 [117] | United Kingdom | Investigation Group | Specific assessment: economic aspects | NA | 1† | Costs and economic evaluation* |  |
| Kolasa and Kozinski (2020) [49], von Huben, A. (2021) [51] | Medical Device Evaluation by CNEDiMTS (Medical Device and Health Technology Evalution Committee), 2019 [118] | France | Government organization | Overall evaluation | By domains | 4† | Health problem and current use of technology; description and technical characteristics of the technology; security; clinical effectiveness* |  |
| von Huben et al (2021) [51] | Medical Services Advisory Committee (AU), 2020 [119] | Australia | Government organization | Overall evaluation | NA | 9† | Health problem and current use of technology; description and technical characteristics of the technology; security; clinical effectiveness; costs and economic aspects; social and patient aspects; legal aspects* |  |
| von Huben et al (2021) [51] | Methodological choices for the clinical development of medical devices, 2013 [120] | France | Government organization | Overall evaluation | NA | 1† | Clinical effectiveness* |  |
| von Huben et al (2021) [51] | Michie, 2017 [121] | United Kingdom and United States | Investigation Group | Overall evaluation | NA | 3† | Security; clinical effectiveness; costs and economic evaluation* |  |
| von Huben et al (2021) [51] | Mohr, 2015 [122] | USA | Investigation Group | Overall evaluation | NA | 1† | Clinical effectiveness* |  |
| von Huben et al (2021) [51] | Murray, 2016 [123] | United Kingdom, Sweden and the United States | Investigation Group | Overall evaluation | By domains | 5† | Health problem and current use of technology; description and technical characteristics of the technology; security; clinical effectiveness; costs and economic evaluation* |  |
| von Huben et al (2021) [51] | Steventon, 2015 [124] | United Kingdom | Non Governmental Organization | Specific evaluation: effectiveness | NA | 1† | Clinical effectiveness* |  |
