# Supplementary figures and images for: Methodological Frameworks and Dimensions to Be Considered in Digital Health Technology Assessment: Scoping Review and Thematic Analysis
Source: J Med Internet Res. 2024 Apr 10;26:e48694. doi: 10.2196/48694 (PMC11043933; doi:10.2196/48694)

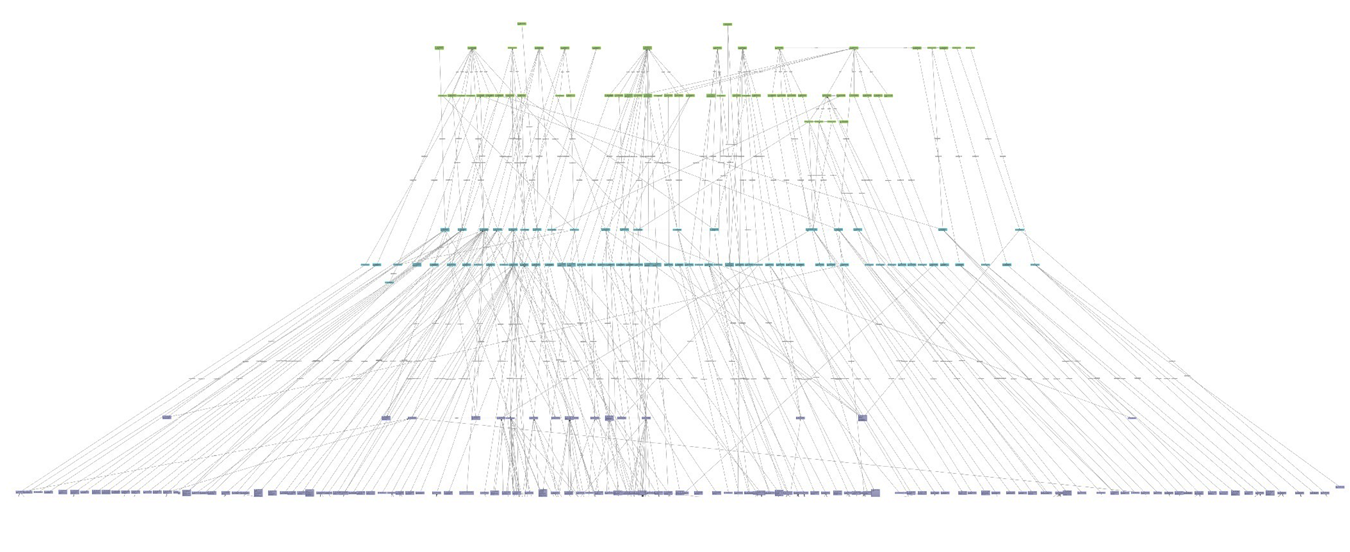

Supplement: Multimedia Appendix 5 [file jmir_v26i1e48694_app5.png]

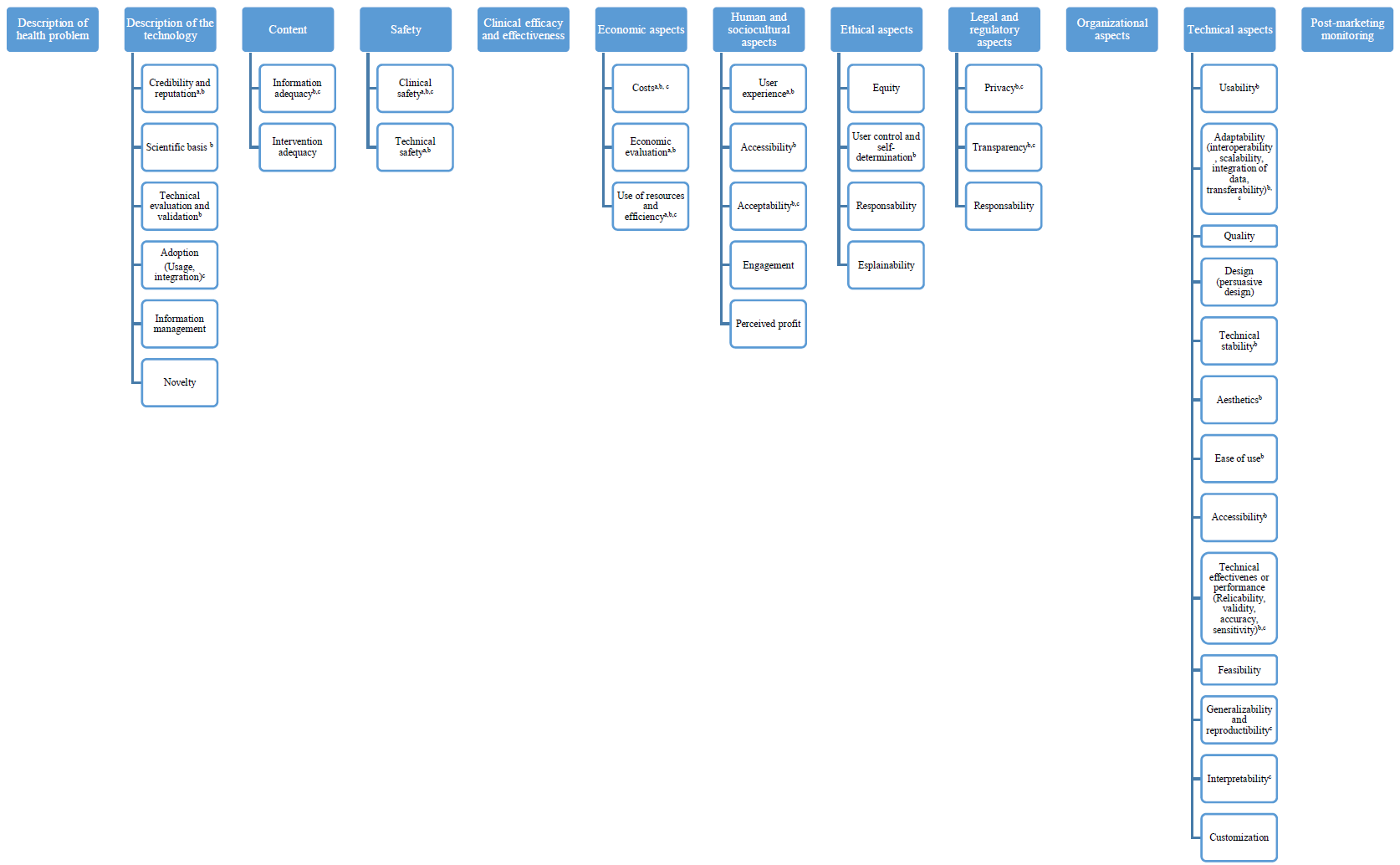

Supplement: Multimedia Appendix 6 [file jmir_v26i1e48694_app6.png]
